# Predictors of responses to immune checkpoint blockade in advanced melanoma

**DOI:** 10.1038/s41467-017-00608-2

**Published:** 2017-09-19

**Authors:** N. Jacquelot, M. P. Roberti, D. P. Enot, S. Rusakiewicz, N. Ternès, S. Jegou, D. M. Woods, A. L. Sodré, M. Hansen, Y. Meirow, M. Sade-Feldman, A. Burra, S. S. Kwek, C. Flament, M. Messaoudene, C. P. M. Duong, L. Chen, B. S. Kwon, A. C. Anderson, V. K. Kuchroo, B. Weide, F. Aubin, C. Borg, S. Dalle, O. Beatrix, M. Ayyoub, B. Balme, G. Tomasic, A. M. Di Giacomo, M. Maio, D. Schadendorf, I. Melero, B. Dréno, A. Khammari, R. Dummer, M. Levesque, Y. Koguchi, L. Fong, M. Lotem, M. Baniyash, H. Schmidt, I. M. Svane, G. Kroemer, A. Marabelle, S. Michiels, A. Cavalcanti, M. J. Smyth, J. S. Weber, A. M. Eggermont, L. Zitvogel

**Affiliations:** 1 INSERM U1015, Gustave Roussy Cancer Campus, Villejuif, 94800 France; 2 University Paris-Saclay, Kremlin Bicêtre, 94 276 France; 3 Gustave Roussy Cancer Campus, Villejuif, 94800 France; 4 Metabolomics and Cell Biology Platforms, Gustave Roussy Cancer Campus, Villejuif, 94800 France; 5 CIC1428, Gustave Roussy Cancer Campus, Villejuif, 94800 France; 6 Gustave Roussy, Université Paris-Saclay, Service de Biostatistique et d’Epidémiologie, Villejuif, F-94805 France; 7 Saint Antoine Hospital, INSERM ERL 1157-CNRS UMR 7203, Paris, 75005 France; 8 Laura & Isaac Perlmutter Cancer Center, New York University Medical Center, New York, NY 10016 USA; 9 Center for Cancer Immune Therapy, Department of Hematology and Oncology, Copenhagen University Hospital, Herlev, DK-2730 Denmark; 10 The Lautenberg Center for General and Tumor Immunology, BioMedical Research institute Israel Canada of the Faculty of Medicine, The Hebrew University Hadassah Medical School, Jerusalem, 91120 Israel; 11 Division of Hematology/Oncology, Department of Medicine, University of California, San Francisco, CA 94143 USA; 12 Department of Immunobiology, Yale School of Medicine, 10 Amistad Street, New Haven, CT 06519 USA; 13 Eutilex, Suite# 1401 Daeryung Technotown 17 Gasan Digital 1-ro 25, Geumcheon-gu, Seoul, 08594 Korea; 14 Section of Clinical Immunology, Allergy, and Rheumatology, Department of Medicine, Tulane University Health Sciences Center, New Orleans, LA 70112 USA; 15 Evergrande Center for Immunologic Diseases and Ann Romney Center for Neurologic Diseases, Brigham and Women’s Hospital and Harvard Medical School, Boston, MA 02115 USA; 16 Department of Dermatology, University Medical Center Tübingen, Tübingen, 72076 Germany; 17 Université de Franche Comté, EA3181, SFR4234, Service de Dermatologie, Centre Hospitalier Universitaire (CHU), Besançon, 25000 France; 18 Department of Medical Oncology, University Hospital of Besancon, 3 Boulevard Alexander Fleming, Besancon, F-25030 France; 19 Clinical Investigational Centre, CIC-1431, University Hospital of Besançon, Besançon, 25030 France; 20 INSERM U1098, University of Franche-Comté, Besançon, 25020 France; 21 Centre Hospitalier Lyon-Sud, Hospices Civils de Lyon and University Claude Bernard Lyon 1, Lyon, 69000 France; 22 Centre de Recherche en Cancérologie de Lyon, Lyon, 69000 France; 23 Department of Pathology, Centre Hospitalier Lyon-Sud, Hospices Civils de Lyon, Lyon, 69000 France; 24 Department of Pathology, Gustave Roussy Cancer Campus, Villejuif, 94800 France; 25 Medical Oncology and Immunotherapy Division, University Hospital of Siena, Viale Bracci, 14, Siena, 53100 Italy; 26 Medical Oncology and Immunotherapy, Department of Oncology, University Hospital of Siena, Instituto Toscano Tumori, Siena, 53100 Italy; 27 Department of Dermatology, University Hospital, University Duisburg-Essen, Essen, Germany & German Cancer Consortium (DKTZ), Heidelberg, D-69120 Germany; 28 Division of Gene Therapy and Hepatology, Centre for Applied Medical Research, Pamplona, 31008 Spain; 29 Oncology Department, University Clinic of Navarra, Pamplona, 31008 Spain; 30 Centro de Investigación cBiomedica en Red de Oncologia, Pamplona, 31008 Spain; 31 Department of Onco-dermatology, CIC Biotherapy, INSERM U1232, CHU Nantes, Nantes, 44000 France; 32 Department of Dermatology, University Hospital Zürich and University of Zürich, Zürich, 8091 Switzerland; 33 Earle A. Chiles Research Institute, Providence Cancer Center, Portland, OR 97213 USA; 34 Sharett Institute of Oncology, Hadassah Medical Organization, Jerusalem, 91120 Israel; 35 Department of Oncology, Aarhus University Hospital, Aarhus, DK-8200 Denmark; 36 INSERM U1138, Centre de Recherche des Cordeliers, Paris, 75006 France; 37 Equipe 11 labellisée par la Ligue contre le Cancer, Centre de Recherche des Cordeliers, Paris, 75006 France; 38 Université Paris Descartes, Sorbonne Paris Cité, Paris, 75006 France; 39 Université Pierre et Marie Curie, Paris, 75005 France; 40 Pôle de Biologie, Hôpital Européen Georges Pompidou, AP-HP, Paris, 75015 France; 41 Department of Surgery, Gustave Roussy Cancer Center, Villejuif, 94800 France; 42 Department of Dermatology, Gustave Roussy Cancer Center, Villejuif, 94800 France; 43 Immunology in Cancer and Infection Laboratory, QIMR Berghofer Medical Research Institute, Herston, QLD 4006 Australia; 44 School of Medicine, University of Queensland, Herston, QLD 4006 Australia

## Abstract

Immune checkpoint blockers (ICB) have become pivotal therapies in the clinical armamentarium against metastatic melanoma (MMel). Given the frequency of immune related adverse events and increasing use of ICB, predictors of response to CTLA-4 and/or PD-1 blockade represent unmet clinical needs. Using a systems biology-based approach to an assessment of 779 paired blood and tumor markers in 37 stage III MMel patients, we analyzed association between blood immune parameters and the functional immune reactivity of tumor-infiltrating cells after ex vivo exposure to ICB. Based on this assay, we retrospectively observed, in eight cohorts enrolling 190 MMel patients treated with ipilimumab, that PD-L1 expression on peripheral T cells was prognostic on overall and progression-free survival. Moreover, detectable CD137 on circulating CD8^+^ T cells was associated with the disease-free status of resected stage III MMel patients after adjuvant ipilimumab + nivolumab (but not nivolumab alone). These biomarkers should be validated in prospective trials in MMel.

## Abstract (web summary)

The clinical management of metastatic melanoma requires predictors of the response to checkpoint blockade. Here, the authors use immunological assays to identify potential prognostic/predictive biomarkers in circulating blood cells and in tumor-infiltrating lymphocytes from patients with resected stage III melanoma.

## Introduction

The recent development of immune checkpoint blockers (ICBs) has rekindled interest in the field of immune cancer therapies^1, 2^. Cancer vaccines^3^, adoptive T cell transfer and CAR T cells^4, 5^, bispecific antibodies^6^, ICBs^7, 8^ and oncolytic viruses^9^ have come of age and many immune agents have recently entered the oncological armamentarium. However, to date, immunotherapy has only been shown to provide durable clinical benefit in a fraction of patients. The recent characterization of multiple immune resistance mechanisms by which tumors can evade the immune system has fueled the development of novel agents that circumvent such limitations, targeting new “immune checkpoints”. It is likely that the use of combination strategies will increase the number of cancer patients that might benefit from immunotherapy^10^. Nonetheless, a number of critical problems remain to be solved. First, the scientific rationale supporting the use of combinatorial regimens needs to be defined. Second, it must be determined whether the future success of immuno-oncology (I-O) will rely on patient stratification in large cohorts or will be personalized to each patient. Depending on tumor characteristics (e.g., PD-L1 or PD-1 expression on tumor cells for anti-PD-1 mAb^11–13^, HMGB1 and LC3B for immunogenic chemotherapy^14^, or tumor microenvironment hallmarks such as IDO expression^15^, macrophage density^16^, tumor-infiltrating lymphocytes [TIL], or Th1 fingerprints^17^), one might envisage more specific and individualized I-O clinical management strategies. Third, predictive immune profiles or biomarkers will need to be validated prospectively to guide I-O utilization in a personalized or stratified manner.

We attempted to address some of these questions in patients with stage III melanoma^18^, given that (i) optimizing adjuvant I-O therapies for metastatic melanoma (MMel) remains an unmet clinical need, (ii) MMel represents a clinical niche for the development of many mAbs and ICBs, (iii) in these patients, metastatic lymph nodes (mLN) are surgically resected, enabling immunological investigations, and (iv) immune prognostic parameters have been recently described in stage III/IV MMel^19, 20^. The tumor microenvironment has a high level of complexity in its regulation. Each checkpoint/co-stimulatory pathway displays an independent mechanism of action. This will require a comprehensive analysis of their mode of action in the tumor microenvironment in any given patient to design appropriate combinatorial approaches and to discover specific biomarkers of response. Herein, we use a systems biology-based approach aimed at defining relevant immunometrics for prediction of an in situ response to cytokines and monoclonal antibodies (mAb) (i.e., agonists and blockers of immune checkpoints) in patients with resected stage III melanoma. In this study, we first describe a suitable “ex vivo metastatic lymph node (mLN) assay”, and through this assay, we demonstrate novel markers for the efficacy of ICB. We then observed, through multivariate analyses performed on eight pooled cohorts including 190 samples of unresectable stage III and IV melanoma, that PD-L1 expression on peripheral blood CD4^+^ and CD8^+^ T cells is prognostic on overall survival (OS) and on progression-free survival (PFS), while in resected stage III melanoma, detectable CD137^+^CD8^+^ peripheral blood T cells predicted a lack of relapse with ipilimumab + nivolumab combination therapy. We conclude that i) the “ex vivo metastatic lymph node (mLN) assay” represents a suitable method to identify biomarkers for ICB and ii) PD-L1 expression on blood CD8^+^ T cells can be a useful marker of resistance to CTLA-4 blockade that needs to be prospectively validated.

## Results

### Functional immunological assays on ex vivo dissociated mLN

The study population of the ex vivo metastatic lymph node (mLN) assay consisted of stage III MMel patients undergoing surgery for lymph node metastases, as previously described^19^. Of these patients, one third presented with more than three involved LN at surgery, 55% had a mutated *BRAF* oncogene, >30% had thyroid dysfunction, and >50% were scheduled to undergo adjuvant therapy. Of primary lesions, 52% were ulcerated. After mechanical and enzymatic digestion of mLN^19^, CD45^–^ cells represented 4–98 ± 4.8% of all cells. The composition of tumor-infiltrating immune cells was analyzed by flow cytometry with gating on live cells in 39 tumor specimens that were paired with autologous peripheral blood cells. Analyses were based on a comprehensive immunophenotyping of 252 parameters (124 in blood and 128 in tumor) per patient, featuring cell type, activation status, naive or memory phenotype, and activating or inhibitory receptors or ligands. We previously found that peripheral blood cell markers were as relevant as TIL immunotypes for OS and PFS of stage III/IV MMel and parameters associated with lymphocyte exhaustion/suppression were associated with greater clinical significance compared to those related to activation or lineage^19^. The next step consisted of analyzing the dynamics of these parameters after incubation with mAbs ± cytokines in 37 patients. Comprehensive assessment of the reactivity of various subsets of infiltrating tumor cells targeting four functional axes, cytokines and their combinations is described (Supplementary Fig. 1 and Supplementary Table 1). A biological response to a given axis was scored “positive” when two independent readouts, reaching a >1.5-fold increase or decrease over two background levels (that of the medium and the isotype control Ab) were achieved. We prioritized fold changes over *p*-values, to reduce false positive candidates (with minimal fold changes, yet very low *p*-values due to small variances). Admittedly, this is an arbitrary choice to select robust effects in the test cohorts but undertook in vivo validation henceforth.

**Fig. 1:**
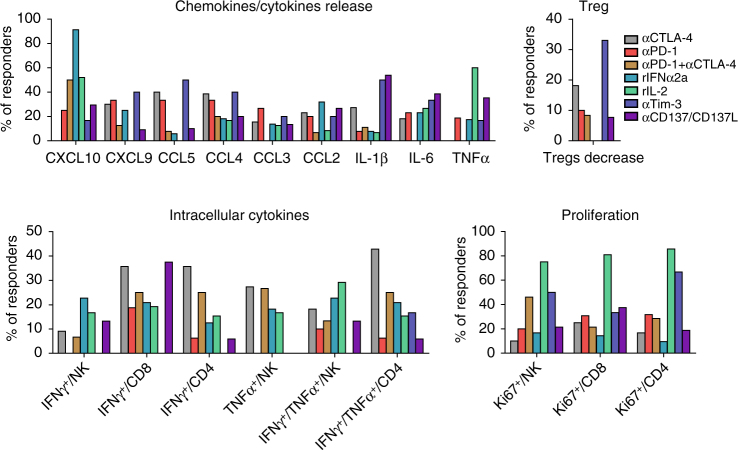
Typification of responses for each axis of stimulation. Summary of Supplementary Figs. 3−5 showing mLN responding to each axis (CTLA-4, PD-1, Tim-3, or combinations, together or with cytokines) by specific immunometrics shared by at least 20% of patients. M&M detail the experimental settings. Briefly, functional assays used flow cytometry determination of early (18–24 h post-stimulation) intracellular cytokine release in T and NK cells, late (day 4–5 post-stimulation) proliferation assays, chemokine and cytokine secretions in the supernatants at 18–24 h. A biological response to a given axis was scored “positive” when two independent readouts, reaching a >1.5-fold increase or decrease over two background levels (that of the medium and the isotype control Ab) were achieved

The “inter-rater agreement” was next evaluated. The inter-individual variability for specimen manipulation, ELISA and flow cytometry analyses was minimal, as demonstrated by two specimens handled by the two first authors independently (Supplementary Fig. 2). Overall, the correlation between the original readings (from ELISA or FACS experiments) was found to be highly satisfactory (Spearman correlation = 0.947/0.857 for patient Pt12/Pt24, number of measurements = 259). When compensating/normalizing to the IgG or control medium, and thus making the data comparable and directly exploitable for the immunometrics scoring, the intraclass correlation remained highly significant (ICC = 0.84 (Pt12) and 0.87 (Pt24)) across all axes considered in the study. Charts depicting the overall relative levels of reactivity in “ex vivo responders” vs. “ex vivo non-responders” for each biological readout and culture condition are presented in Supplementary Figs. 3−5. As a positive control, ex vivo IL-2 stimulation of mLN frequently induced T and NK cell proliferation, as well as cytokine release mostly by NK cells (Fig. 1, Supplementary Fig. 3). Additionally, ex vivo stimulation with rIFNα2a led to high CXCL10 release (Fig. 1 and Supplementary Fig. 3). mLN responding ex vivo to PD-1 blockade exhibited T-cell proliferation and chemokine release (CCL2, CCL4, and CXCL10) in 20–33% of cases (Fig. 1 and Supplementary Fig. 4). mLN responding ex vivo to CTLA-4 blockade demonstrated polyfunctional T-cell activation (in 28 and 42% of responders for CD8^+^ and CD4^+^ T cells, respectively) and chemokine release (CCL4, CCL5, and CXCL9 in 30–40% of responders) (Fig. 1 and Supplementary Fig. 4). mLN responding ex vivo to CTLA-4/PD-1 co-blockade typically showed NK cell proliferation (in 46% of responders) and CXCL10 release (in 50% cases) (Fig. 1, Supplementary Fig. 5). Anti-Tim-3 mAb led to NK and CD4^+^ T-cell proliferation, inflammatory cytokines and CCL4/CCL5 production in 2–3 out of 6 responding lesions (Fig. 1, Supplementary Fig. 5). mLN responding ex vivo to CD137/CD137L stimulation exhibited CD8^+^ T-cell proliferation accompanied by IL-1β, IL-6, and TNFα release in 30−37% of responding lesions (Fig. 1, Supplementary Fig. 5).

**Fig. 2:**
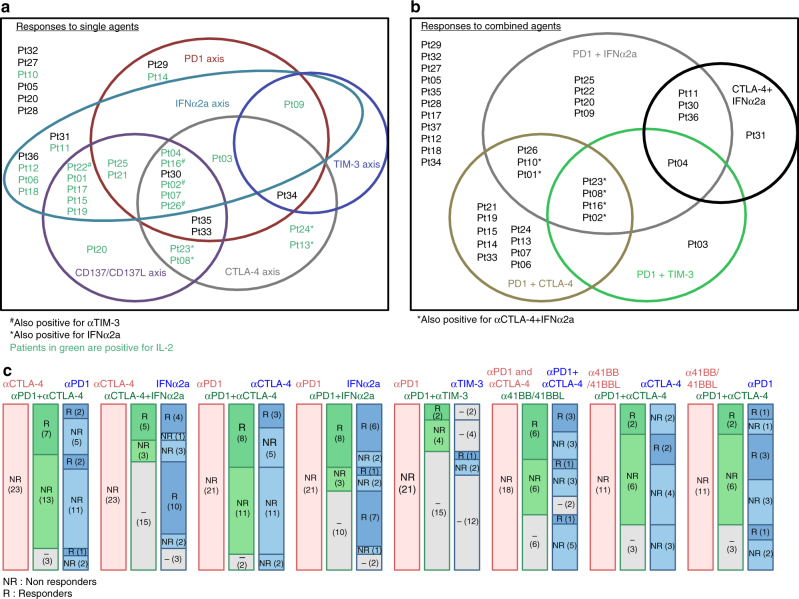
Global representation of the patterns of responses to individual or combined stimulations for 37 MMel. **a**, **b** Venn diagram representing each stimulating axis alone **a** or in combination **b** per circle, patients being identified by letters and numbers. **c** Frequencies of patient lesions that failed to respond to a given axis (*first bar*, in *red*) but could exhibit significant responses to alternative axis of stimulation. (*second and third bar* in *green* and *blue*). For instance (*very left bars*), in the non-responding lesions (NR) to anti-CTLA-4 Ab, we annotated the percentages that could respond (or not) to anti-CTLA-4 + anti-PD-1 Ab co-blockade (in *green*), among which some of them could respond (or not) to anti-PD-1 Ab alone (in *blue*). The detailed patterns of responses feature in Supplementary Table 1. In *gray boxes*: Not Done

The Venn diagrams detailing the patterns of immune reactivities are depicted in Fig. 2. The proportions of mLN “ex vivo responding” to at least one I-O axis were ~ 30–50 and 50–60% for mAb combinations (Supplementary Table 1, Fig. 2a, b). The proportion of mLN “ex vivo responding” to both anti-CTLA-4 and anti-PD-1 mAb separately was 11/37 (30%), among which 45% failed to respond to concomitant blockade (Supplementary Table 1). Sixty percent (17/28) of mLN were “ex vivo responders” to agonistic anti-CD137/anti-CD137L mAb among which 35% (6/17) failed to respond to any of the classical ICBs (anti-CTLA-4 or anti-PD-1 mAbs) (Supplementary Table 1). The likelihood of “ex vivo response” to any alternate ICB or mAb combination in cases failing to respond to any one monotherapy or combination therapy is depicted in Fig. 2c. Altogether, our ex vivo mLN assay is a feasible test potentially allowing a diagnosis of prediction of ex vivo response to 11 conditions of stimulation.

### Predictive biomarkers of resistance to CTLA-4 blockade

**Fig. 3:**
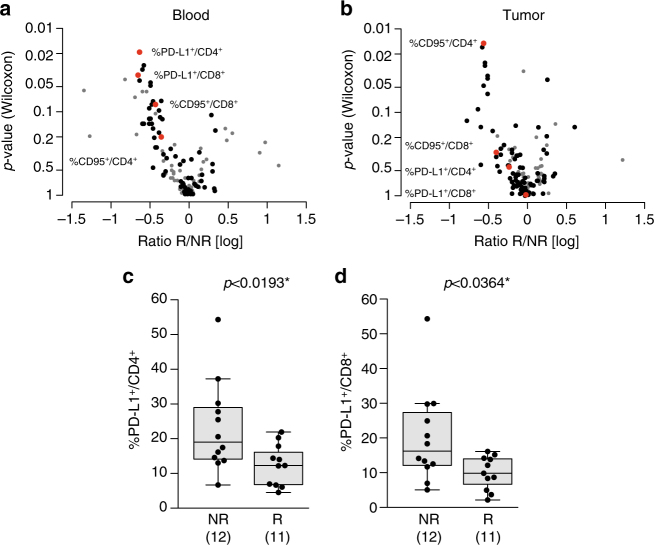
PD-L1 expression on T cells predicts reactivity to ipilimumab in the “ex vivo mLN assay”. **a**, **b** Display of the Wilcoxon rank-sum test *p*-values vs. the log transformed ratio between responders (R) and non-responders (NR) to anti-CTLA-4 Ab in blood **a** or tumor **b** samples. Each *dot* represents one marker; selected biomarkers are shown in *red* while biomarkers with very low level of expression are shown in *gray*. **c**, **d** Expression levels of PD-L1 on blood CD4^+^ **c** and CD8^+^ **d** T cells in patient lesions responding (R) or not (NR) to the ex vivo mLN assay using a stimulation with anti-CTLA-4 mAb. Each *dot* represents one patient. The absolute numbers of patients are indicated in parentheses in both groups. Graphs were analyzed by Wilcoxon rank-sum test. Box and whiskers plots are represented from the corresponding distribution **c**, **d**

We next addressed whether predictive biomarkers of a functional response obtained in the ex vivo mLN assay could be inferred from the 779 blood/tumor parameters. Multivariate analyses by means of PCA on all available information are routinely performed to assess variance structure in the data and in particular for quality control purposes to identify potential outlying samples and/or features. This analysis did not reveal obvious clustering of the samples and their distribution on the first few components could not be significantly associated to any clinical parameters. Very few statistically significant immune parameters predicting responses to CTLA-4 blockade could be found (Fig. 3a, b and Supplementary Fig. 6a, b).

The strongest predictive biomarkers of ex vivo resistance to CTLA-4 blockade were elevated PD-L1 expression on circulating CD4^+^ T cells (Fig. 3a, c, AUC = 0.79, *p* < 0.01, (Wilcoxon rank-sum test)) and CD8^+^ T cells (Fig. 3a, d, AUC = 0.76, *p* < 0.03 [Wilcoxon rank-sum test] without adjustment for multiple comparisons) but not on the tumor-infiltrating lymphocytes in lymph node melanoma metastases (Fig. 3b and Supplementary Fig. 6c, d). Hence, pending further evaluation (see below) this potential biomarker may be relevant to circulating T lymphocytes but not to the tumor immune infiltrate. Other potential biomarkers such as CD95 expression (best significance in blood for CD8^+^ T cells, *p = *0.08, [Wilcoxon rank-sum test] and in tumors for CD4^+^ T cells, *p < *0.01 [Wilcoxon rank-sum test], Supplementary Fig. 6e−h) were also selected in the model. Of note, CD95 membrane expression on CD4^+^ T cells was dominant in Treg and chronically activated CD4^+^ T cells as well as terminally differentiated effector CD8^+^ T cells (but not naive T cells, Supplementary Fig. 7a, b), and highly correlated with HLA-DR and PD-1 expressions (Supplementary Fig. 7c, d). Additionally, although retained in the statistical analyses, some biomarkers were not considered further due to their weak detectability (<2% expression) and low robustness of their flow cytometric analyses.

Although the aforementioned correlations between immune parameters and in vitro/ex vivo response patterns were weak (in the sense that they were only borderline significant), we hypothesized that PD-L1 and/or CD95 on circulating CD4^+^ and CD8^+^ T cells might predict resistance to CTLA-4 blockade in MMel. We therefore decided to investigate these parameters in another patient cohort to correlate these immune parameters with in vivo responses to CTLA-4 blockade in MMel.

**Fig. 4:**
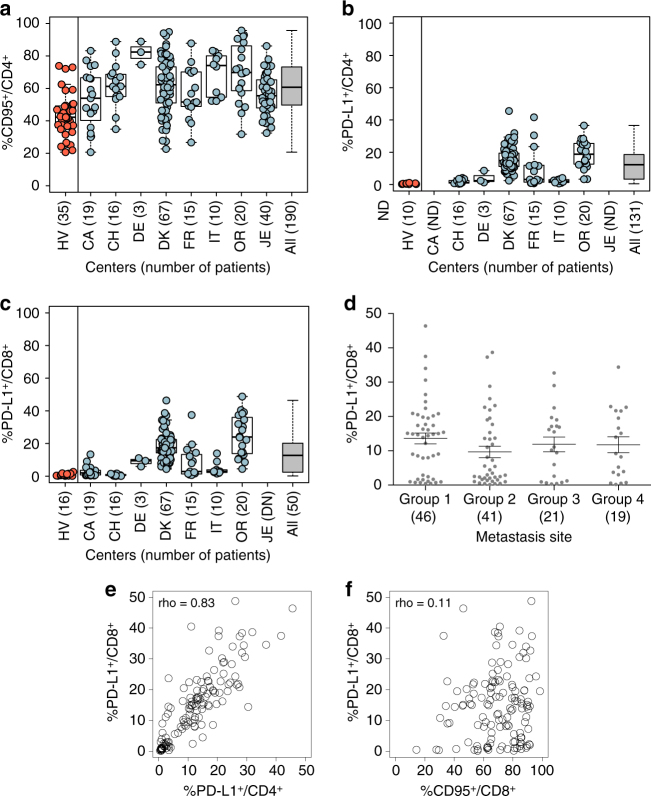
Melanoma patients express higher levels of PD-L1 on circulating T cells than healthy volunteers. **a**−**c** Percentages of CD95 **a** and/or PD-L1^+^ **b**, **c** cells among blood CD4^+^ **a**, **b** or CD8^+^ **c** T cells, respectively, at baseline prior to ipilimumab. Flow cytometric assessments of the proportions of blood CD3^+^CD4^+^ or CD8^+^ cells expressing PD-L1 after thawing in eight cohorts of MMel (*right columns*) except CA and/or JE (not assessed), as well as 10–35 healthy volunteers (in *red*, *left column*). Each *dot* represents one healthy volunteer or patient. Mean and s.e.m. are represented along with the box plots for each cohort described in Supplementary Table 2. **d** Expression of PD-L1 on CD8^+^ T cells according to the metastatic sites: 1 (skin, lymph node and lung metastases only), 2 (visceral metastases, soft tissues +/− group 1), 3 (bone metastases and+/− groups 1 and/or 2) and 4 (brain metastases and others). **e**, **f** Spearman correlation between PD-L1^+^/CD8^+^ and PD-L1^+^/CD4^+^ or CD95^+^/CD8^+^ with rho index; each *dot* representing one patient. Box and whiskers plots are represented from the corresponding distribution **a**−**c**. Mean + s.e.m. **d**

Ipilimumab not only improves overall survival in stage IV MMel but also impacts overall survival, recurrence-free survival and distant metastasis-free survival in resected high-risk stage III melanoma^21, 22^. Based on the results of the “ex vivo mLN assay”, we retrospectively evaluated the previously selected biomarkers PD-L1 and CD95 on clinical response and survival outcomes (overall survival and progression-free survival). Data were available for 190 unresectable stage III and IV MMel patients treated with 3 mg/kg (in 90% cases) of ipilimumab in eight cohorts from different centers. The median follow-up is 30 months (95% Confidence Interval (CI): 26–34). Patients’ characteristics are presented in Supplementary Table 2. PD-L1 and CD95 were evaluated retrospectively at diagnosis in whole blood or peripheral blood mononuclear cells (PBMCs) (after density gradient separation of cells) by flow cytometry gating on CD4^+^ and/or CD8^+^ T cells using a standardized methodology validated for all centers (either performed by our laboratory, after thawing of cryopreserved cells or by the investigators themselves using our antibodies and procedures). CD95 expression levels were higher in MMel compared with healthy volunteers (HV) in blood T cells (Fig. 4a). Although variable according to centers and individuals, PD-L1 expression levels were highly detectable in circulating CD4^+^ (Fig. 4b) and CD8^+^ (Fig. 4c) T cells in stage III/IV MMel patients, while remaining below the threshold of confidence in HV (Fig. 4b, c). Additionally, no significant expression was observed between PD-L1 expression on CD8^+^ T cells and either the LDH status (*p* = 0.71, [Student’s *t*-test] comparing center-specific high vs. low LDH levels) or the metastases localization (Fig. 4d). On the other hand, PD-L1 expression on CD8^+^ and CD4^+^ T cells are highly correlated (rho = 0.83, Fig. 4e) but not with CD95 expression (rho between 0.01 and 0.12, e.g., Fig. 4f).

**Fig. 5:**
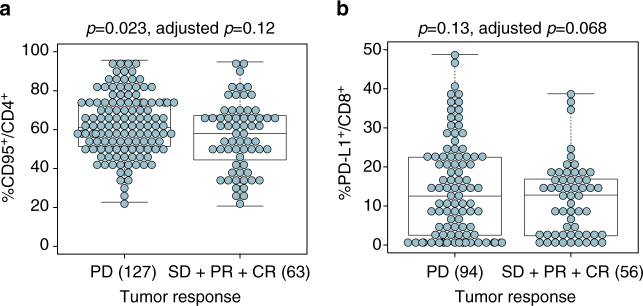
Predictive values of PD-L1^+^/CD8^+^ and CD95^+^/CD4^+^ for RR to ipilimumab. **a**, **b** Statistical analyses of the clinical relevance of CD95 expression on CD4^+^ T cells as well as PD-L1 on CD8^+^ T cells according to RR (separating PD from SD, PR, or CR) were performed in univariate and multivariate regression assays. Each *dot* represents one patient. The box plots indicating the mean and s.e.m. of values for the binary separation are depicted (cf Supplementary Table 4). The absolute numbers of patients are indicated in all groups in parentheses. Box and whiskers plots are represented from the corresponding distribution **a**, **b**

To avoid reducing the power of the statistical analysis, PD-L1 and CD95 biomarkers have been considered on a continuous scale. First, the tumor response evaluated at 3 months was categorized into 4 groups: progressive disease (PD, *n* = 127, 67%), stable disease (SD, *n* = 31, 16%), partial response (PR, *n* = 18, 9%) and complete response (CR, *n* = 14, 7%) (Supplementary Table 2). The chosen binary outcome for the logistic regression model was: PD (*n* = 127, 67%) vs. SD + PR + CR (*n* = 63, 33%). Supplementary Table 3 shows the impact of clinical covariates on tumor response and survival endpoints (PFS and OS). Even if some of the presented clinical covariates were not significant in the univariate analysis, we kept them all in the final model as they are recognized as potential prognostic factors. Hence, final models were stratified on the centers and adjusted for LDH (“low or high”, meaning below or above the normal value for each clinical center), previous chemotherapy (“yes” or “no”), previous immunotherapy (“yes” or “no”), previous protein kinase inhibitor (“yes” or “no”), gender (“male” or “female”), age (continuous scale) and tumor stage (III or IV). Regarding the clinical response at 12 weeks, the expression of CD95 on CD4^+^ T cells was observed to be prognostically associated in univariate analysis (Fig. 5a, *p* = 0.023 [Cox modeling]), however, neither CD95 nor PD-L1 on blood CD4^+^ and CD8^+^ T cells was observed to be significant in the multivariate analyses (Supplementary Table 4). Of note, the association of PD-L1^+^/CD8^+^ on clinical response is border line (Fig. 5b, *p = *0.068 [Cox modeling] in multivariate analyses).

**Fig. 6:**
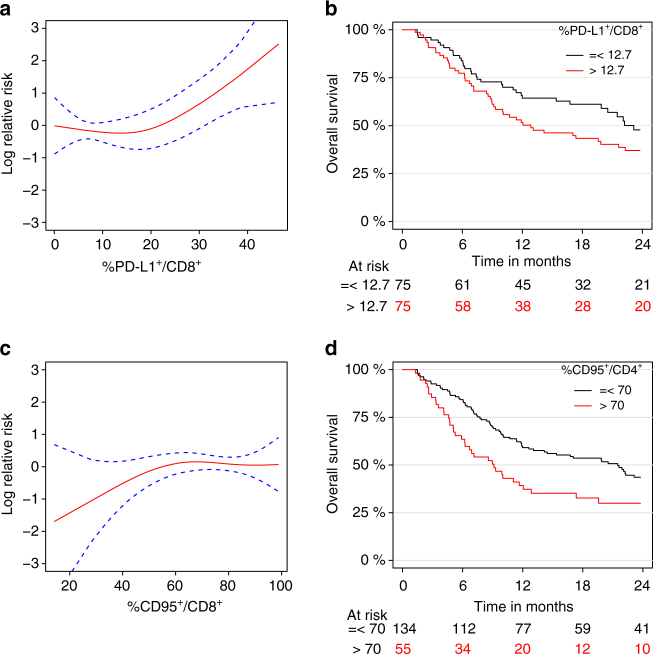
Relative risk of death according to PD-L1 or CD95 on T cells. Overall survival is fulfilled for *n* = 189 patients including 121 events. **a** Graphical visualization of the log relative risk according to the expression of PD-L1 on CD8^+^ T cells (*p* = 0.011 in the multivariate analysis, Supplementary Table 5). The *dashed blue lines* represent the 95% confidence intervals. **b** Kaplan−Meier OS curves segregating the whole cohort according to the median value (i.e., 12.7%) for the PD-L1 expression on blood CD8^+^ T cells at baseline. **c** Graphical visualization of the log relative risk according to the expression of CD95 on CD8^+^ T cells (*p* = 0.33 in the multivariate analysis, Supplementary Table 5). The *dashed blue lines* represent the 95% confidence intervals. **d** Kaplan−Meier OS curves segregating the whole cohort according to a cutoff value of 70% for the CD95 expression on blood CD4^+^ T cells at baseline

Next, we analyzed the impact of those biomarkers on PFS (in 169 MMel including 143 events) and OS (in 189 MMel including 121 events). The 2-year PFS and OS were 17% (95% CI: 11–23%) and 40% (95% CI: 32–47%), respectively (Supplementary Fig. 8). Based on the univariate analysis, the highest prognostic clinical covariates on PFS was history of protein kinase inhibition (PKI), while LDH and previous chemotherapy/radiotherapy or PKI were associated with the OS (Supplementary Table 3). PD-L1 expression on circulating CD4^+^ T cells and to a lesser extent on CD8^+^ T cells were prognostic on PFS after CTLA-4 blockade (Supplementary Table 5, *p = *0.009 [Kaplan−Meier methods] for PD-L1^+^CD4^+^ and *p = *0.056 [Kaplan−Meier methods] for PD-L1^+^CD8^+^ in multivariate analyses). Similarly, PD-L1^+^CD8^+^ T cells was observed to be prognostic on OS (Fig. 6a, b, *p = *0.011 [Kaplan−Meier methods] in multivariate analyses) outperforming PD-L1^+^CD4^+^ T cells (*p = *0.081 [Kaplan-Meier methods] in multivariate analyses) (Supplementary Table 5). For both PFS and OS, the higher is the expression of PD-L1, the higher the risk. On the other hand, no significant association was highlighted between the CD95 expression (on both circulating CD4^+^ and CD8^+^ T cells) and survival endpoints (Supplementary Table 5). Even if no significant association was observed, Fig. 6c illustrates a protective tendency for patients with extremely low expression level of CD95^+^ on CD8^+^ T cells. We also investigated a cutoff value of 70% for the expression of CD95 on CD4^+^ T cells (Fig. 6d) but did not find association on OS in multivariate analysis. It should be noted that the frequency of circulating PD-L1^+^CD8^+^ T cells impacted OS more than PFS with a hazard ratio ± 95 confidence interval for OS = 1.053 (1.012–1.096) and for PFS = 1.032 (0.999–1.065), in line with the fact that immune-related parameters generally have a larger influence on OS than on PFS^23–25^.

Altogether, our data indicate that PD-L1^+^ expression especially on CD8^+^ T cells is associated with CTLA-4 blockade (mostly 3 mg/kg) for OS and borderline for RR and PFS in unresectable stage III and IV MMel.

### Predictive biomarkers of response to CTLA-4 + PD-1 co-blockade

**Fig. 7:**
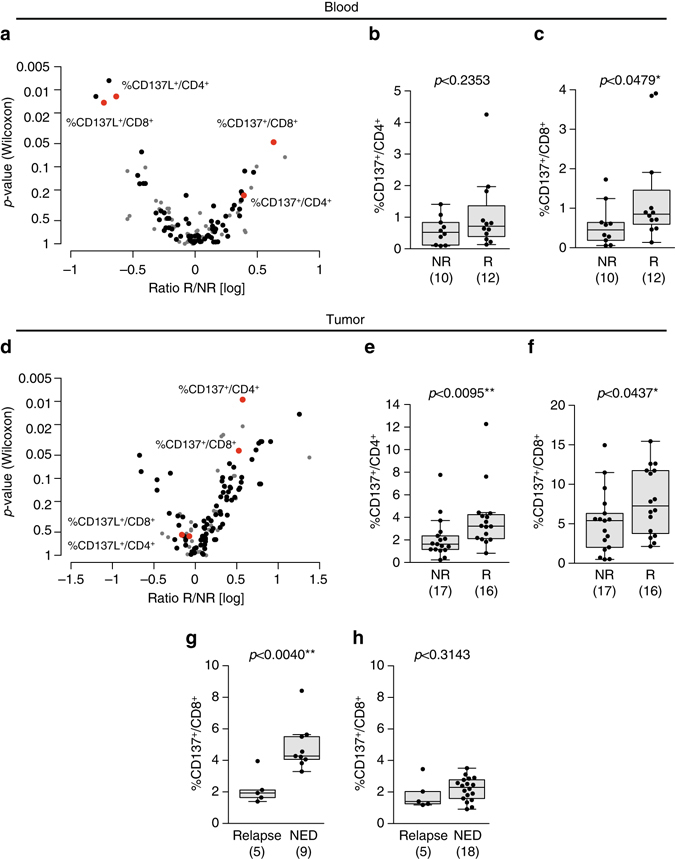
CD137 expression on blood CD8^+^ T cells predicts sensitivity to the combination of anti-CTLA-4+ anti-PD-1 Ab. **a**, **d** Display of the Wilcoxon rank-sum test *p*-values vs. the log transformed ratio between responders (R) and non-responders (NR) to anti-CTLA-4 + anti-PD-1 Abs in the blood **a** and tumor **d** in the ex vivo mLN assay. Each *dot* represents one marker; selected biomarkers are shown in *red* while biomarkers with low level of expression are shown in *gray*. **b**−**f** Expression levels of CD137 in blood **b**, **c** and tumor bed **e**, **f** of CD4^+^ **b**, **e** and CD8^+^ T **c**, **f** cells, respectively, in patient lesions responding (R) or not (NR) in the ex vivo mLN assay anti-CTLA-4 + anti-PD-1 mAbs stimulatory condition. Each *dot* represents one patient. The absolute numbers of patients are indicated in both groups in parentheses. Graphs were analyzed by Wilcoxon rank-sum test. **g**, **h** Prospective analysis of CD137 expression on T cells prior to enrollment in a Phase II trial of high risk stage IIIc/IV MMel patients. Distributions of CD137 expression on blood CD8^+^ T cells at diagnosis prior to PD-1/CTLA-4 co-blockade **g** or PD-1 blockade **h** across patient groups stratified based on progression (relapse or no evidence of disease [NED]) with a median follow-up of 13 months post enrollment in adjuvant therapy for stage III/IV resected MMel. Each *dot* represents the flow cytometry analysis value of CD137 expression compared with an isotype control mAb prior to adjuvant therapy. Reported *p*-values are obtained from the Wilcoxon rank-sum test. Box and whisker plots are represented from the corresponding distribution **b**, **c**, **e**−**h**

The regimen of ipilimumab and nivolumab has demonstrated impressive clinical benefit in MMel (objective response rate (ORR) >60% with a PFS >11 months), but is also associated with a high rate of immune related adverse events (>50% grade 3–4 events)^26^. This supports further investigation into biomarkers which may predict which patients may derive the most benefit to spare primarily resistant patients the toxicity of the treatment. Given that the proportion of mLN that respond to both anti-CTLA-4 and anti-PD-1 mAb separately was 11/37 (29%), among which 45% failed to respond to combined blockade (Supplementary Table 1, Fig. 2), we hypothesized that predictive biomarkers of response to this combination would have different immunometrics than those identified for anti-CTLA-4 blockade. Again, few immune parameters in blood and tumors were found to be associated with functional responses to co-blockade (Fig. 7a–d and Supplementary Fig. 9a, b). The two superior immunometrics retained in the assay of 779 variables were the expression levels of CD137/4-1BB on circulating CD4^+^ and CD8^+^ T lymphocytes (Fig. 7b, c). Detectable expression levels of CD137 on blood and tumor CD8^+^ T lymphocytes (and to a lesser extent in CD4^+^ T cells) at diagnosis were correlated to response to combined anti-PD-1/CTLA-4 mAbs (Fig. 7b, c, e, f). Based on these findings obtained in blood and tumor, we hypothesized that CD137 critically impacted sensitivity to CTLA-4/PD-1 co-blockade in MMel.

To assess the predictive value of CD137 expression on circulating CD8^+^ T cells at baseline for clinical benefit from the combination of ipilimumab and nivolumab, we analyzed this parameter in PBMCs obtained from a phase II adjuvant trial assessing the efficacy of nivolumab and ipilimumab combination therapy in resected stage IIIc and IV MMel. The median follow-up of this study was 13 months. The expression levels of CD137 on circulating CD8^+^ T cells at baseline in this cohort of patients was within the range of those described above in patients with metastatic disease (Fig. 7g, h). Interestingly, stage III MMel patients with resected high risk disease who did not relapse after combination therapy expressed much higher levels of CD137 on their circulating CD8^+^ T cells at enrollment in the Phase II adjuvant trial, compared with the levels in patients who had a relapse (*p = *0.004 [Wilcoxon rank-sum test]) (Fig. 7g).

Of note, low CD137 expression on CD8^+^ T cells did not predict relapse in patients with high-risk resected melanoma treated with nivolumab alone as anticipated from our correlative matrices (Fig. 7h). To analyze which biomarker was best associated with clinical outcome to PD-1 blockade, we returned to the ex vivo mLN assay described above. The best immunometrics obtained on circulating T cells and retained in the model of 779 variables were (i) PD-1 expression levels on CD4^+^ T cells, (ii) the ratio between CD8^+^ lymphocytes and CD127^low^CD25^high^ CD4^+^ Treg cells, (iii) PD-L1 expression on CD4^+^ and CD8^+^ T cells as shown for ipilimumab (Supplementary Fig. 10a). Indeed, higher expression levels of PD-1 (>20%) in circulating CD4^+^ T cells at diagnosis was associated with the likelihood to respond in the ex vivo mLN functional assays using anti-PD-1 mAb (but not other mAbs; *p* < 0.02 [Wilcoxon rank-sum test], AUC = 0.75) (Supplementary Fig. 10b). A CD8^+^ T cell/Treg ratio >5 also tended to predict ex vivo reactivity of mLN to PD-1 blockade (but not to another I-O axis; *p* < 0.06 [Wilcoxon rank-sum test], AUC = 0.73) (Supplementary Fig. 10c). Similar to CTLA-4 blockade, lower expression levels of PD-L1 on circulating CD4^+^ and CD8^+^ T cells were associated with ex vivo reactivity of PD-1 blockade (Supplementary Fig. 10d).

Altogether, this study demonstrates that the ex vivo mLN assay, as well as the preselected biomarkers of response or resistance to mAbs may identify patients likely to respond to, or fail to benefit from the proposed therapy.

## Discussion

We describe new predictive biomarkers of response to CTLA-4 blockade and to effective but potentially toxic combination therapy composed of anti-CTLA-4 + anti-PD-1 mAbs. These results are based on a functional method called “the ex vivo mLN assay”, capable of assessing the reactivity of metastatic lymph node infiltrating immune effectors (T and NK cells) during stimulation with various ICB or agonistic mAbs and their combinations. This was coupled with a paired blood and tumor immune profiling of mLN in stage III MMel with the intention of correlating immune fingerprints with clinical parameters^27, 28^. We elucidated the relevance of PD-L1 expression on circulating T cells for the prediction of resistance to ipilimumab, alone or combination with IL-2 or GM-CSF. Moreover, our study shows that detectable levels of CD137 on circulating CD8^+^ T cells after LN or metastatic resection in stage IIIc and IV melanoma tends to predict longer PFS for the anti-CTLA-4 + anti-PD-1 co-blockade.

The ex vivo mLN assay was feasible for almost all mLN specimens containing at least 10^7^ cells (37/46 were successfully performed and contained enough cells for the “ex vivo mLN assay”). Of note, this method could be downscaled to the size of a biopsy if only 1 or 2 mAbs had to be tested. The method is also reliable in that the two negative controls used (18–24 h or a 4–5 day incubation in the absence of stimulus or in the presence of Ig control mAb) allow the basal assessment of T cell functions to be determined^19^ with low non-specific backgrounds. The high dose rIL-2 and rIFNα2a positive controls almost invariably triggered effector (and Treg) proliferation and CXCL10 release, respectively, in all patients. We show that this method can analyze important dynamic T and NK cell parameters relevant to effector functions against cancer, such as proliferation and release of Th1 cytokines, as well as proportions of Tregs in the co-culture system. Cytokine and chemokine release could be considered as surrogate markers for effector cell trafficking or homing to inflammatory sites.

It should be noted that the biomarkers that we chose to validate and that were initially characterized in the discovery cohort were chosen without adhering to the usual practice of correcting for multiple comparisons. Rather, we preferred to include candidate biomarkers based on raw (univariate) *p-*values of <0.05 that reflected results that could be robustly quantified and that appeared biologically relevant with fold changes between positive and negative controls of >1.5 for a given axis. It is only by testing additional cohorts of patients who received ipilimumab in vivo that these candidate biomarkers acquired a potential significance.

The findings from our study indicate that the mLN reactivity to immunomodulators is specific for each patient since (i) a precise and specific pattern of immune activation for each mAb or their various combinations across patients was not possible, in contrast to generalizable responses to rIL-2 or rIFNα2a; (ii) each individual patient exhibited a specific pattern of response to the panel of stimulatory agents. Interestingly, our long-term expertise with this ex vivo tumor restimulation assay underscores the relevance of the tumor microenvironment in dictating the functional outcome. Indeed, GIST responded best to anti-IL-10 or anti-TRAIL mAbs or rIFNα2a, rather than to anti-PD-1 or anti-CTLA-4 mAbs^29^.

Our study also uncovers, for the first time, two biomarkers of resistance or response to I-O regimens: ipilimumab alone or combined with PD-1 blockade. Herein, we found that the most prominent markers predicting response to such regimens were not the obvious candidates. PD-L1 (and not CTLA-4) on T cells was found crucial for the prediction of resistance to anti-CTLA-4 mAb, whereas CD137 expression on circulating CD8^+^ T cells appears a promising predictor of long term (>13 months) relapse-free survival mediated by the combination of anti-PD-1 and anti-CTLA-4 mAbs in the adjuvant setting. This should be investigated further in the metastatic setting and validated in additional adjuvant patients.

Most previous biomarker studies with PD-1/PD-L1 antibodies have focused on the prognostic significance of PD-L1 (and/or PD-L2) expression on tumor cells or myeloid cells of the TME. Expression of both PD-L1 and PD-L2 significantly correlated with increasing densities of immune cells in the tumor specimens and with immunotype. Positive PD-L2 expression alone or combination with PD-L1 expression, was associated with improved overall survival^30^. High PD-L1 expression on melanoma were found predominantly in regions of abundant inflammation or TIL infiltrates, even in sanctuaries like brain metastases^31^, but it failed to predict responses to ICB in MMel. To our knowledge, this is the first comprehensive analysis of the predictive role of PD-L1 expression on peripheral blood T cells in melanoma. This expression might reflect the chronic exposure to type 1−type 2 IFNs in the TME in recirculating TILs^20^, as already reported in tumor cells themselves^32^.

In contrast, we could not identify a significant association of the CD95/CD4 biomarker neither with OS nor PFS in multivariate analyses in these cohorts of MMel. However, given its biological relevance^33–43^, higher levels (>70%) detected in MMel (compared with HV), and co-expression of a variety of inhibitory receptors on CD95^+^CD4^+^ T cells (such as PD-1, and HLA-DR, Supplementary Fig. 7), it might still be interesting to consider this biomarker in prospective studies using cutoff values >70%.

The combination of ICBs ipilimumab and nivolumab has been FDA-approved for first-line treatment of unresectable MMel. This approval followed the results of CheckMate 067^26^–069^44^, trials where the combination of ipilimumab and nivolumab outperformed each single agent alone in terms of response rates and PFS. Additionally, recently published data in non-small cell lung cancer patients have shown promising results for the combination of anti-PD-L1 and anti-CTLA-4 mAbs in a phase 1b clinical trial^45^. Hence, such combinations may be integrated into the ever-changing melanoma treatment algorithm, and will most likely be extended to other malignancies sensitive to PD-1 blockade. However, drug-related adverse events of grade 3 or 4 have been reported in 54% of patients receiving ipilimumab/nivolumab combination therapy, as compared with 24% of patients receiving ipilimumab monotherapy^46, 47^. Such immune-related adverse events are generally reversible with immunosuppressive medications. Given the efficacy and relative safety of nivolumab alone, finding a predictor of response to such potentially toxic combinations is an urgent unmet clinical need. Here, we propose a biomarker of response to ipilimumab + nivolumab: the presence of detectable levels of CD137 on blood CD8^+^ T cells, which appears to be significantly associated with a lack of relapse in resected high-risk, treatment-naive stage III MMel. This novel biomarker is based on the following data: (i) circulating T-lymphocytes expressing CD137 could be found in the blood of patients with no evidence of disease with a median follow-up of 13 months who received the combination in an adjuvant setting (and not in those where nivolumab was administered alone); (ii) the finding from the ex vivo mLN assay that CD137 is upregulated in CD4^+^ and CD8^+^ TILs in lesions found to be “responding” to ex vivo stimulation with the combination of anti-PD-1 + anti-CTLA-4 mAbs (and not to anti-PD-1 mAb or to other combinatorial regimens). It is therefore conceivable that this combinatorial stimulation leads to the engagement of the CD137/CD137L co-stimulatory pathway, required for T cell fitness and recirculation in the blood of responders^20^. However, this pathway did not appear responsible for tumor rejection mediated by the combination regimen in mouse models, although the addition of an agonistic CD137 mAb to the combination therapy further delayed tumor outgrowth in a therapeutic MCA-induced sarcoma model (MJS, unpublished data). This data confirm a previous study performed in mouse ovarian carcinomas, where agonistic anti-CD137 mAb augmented the impact of anti-PD-1 + anti-CTLA-4 mAb therapy^48^.

These novel predictive immunometrics add to the long list of putative biomarkers potentially relevant for ICB therapies. Our previous experience suggested that high LDH levels, CXCL11 and sCD25 concentrations in the serum negatively predict time to progression in ipilimumab-treated stage IV MMel^49–52^, whereas CLA (Cutaneous Lymphocyte Antigen) expressing CD8^+^ T_EM_ represent a pharmacodynamic signature of sensitivity to CTLA-4 blockade^20^. HLA subtype^53^, genetic polymorphisms^54^, and absolute lymphocyte counts^55^ have not been validated as immunotherapy biomarkers. A number of alternative parameters such as high baseline levels of Foxp3 and IDO expression^54^, increased TILs and Th1 cells at baseline^56^, MDSC numbers^50, 57, 58^, T cell ICOS expression as pharmacodynamic markers^59^, and (more recently) high mutational load and neoantigen landscape^60, 61^, have yet to be prospectively studied as biomarkers for the efficacy of immunotherapy for melanoma.

A number of biomarkers of response to anti-PD-1/PD-L1 mAbs have been considered promising for future prospective validation. For example, selective CD8^+^ T cell tumor infiltration (often correlated with PD-L1 expression) and their distribution at tumor invasive margins preceding PD-1 blockade appear to predict ORR in stage IV melanoma^62–64^. Similarly, the immunohistochemical determination of PD-L1 expression (although lacking a standardized methodology and subject to variable expression depending on timing and biopsy sites) may guide the choice between PD-1 blockade vs. CTLA-4 + PD-1 co-blockade^63–65^. A high mutational load is also associated with clinical responses to the PD-1 regimen^66^. Moreover, high relative eosinophil count, and lymphocyte count, low LDH and absence of metastasis other than soft-tissue or lung at baseline are associated with a favorable OS in patients treated with pembrolizumab^67^. Whether the proposed blood biomarkers identified in our study (PD-1 expression on CD4^+^ T cells, PD-L1 expression on CD4^+^ and CD8^+^ T cells, or the CD8^+^ T cell/Treg ratio in blood) would also be useful to predict the efficacy of PD-1 blockade remains to be elucidated in large retrospective and then prospective cohorts. Our findings suggest that prospective ICB adjuvant trials in stage III−IV MMel could be personalized based on (i) ex vivo mLN assays or (ii) blood biomarkers capable of predicting such response. Obviously, further clinical trials are necessary to validate this prediction.

## Methods

### Experimental design

In a cohort of stage III MMel patients, we previously reported the immune parameters that were significantly associated with outcome^19^. We established an ex vivo assay based on the reactivity of immune cells from 37 dissociated metastatic lymph nodes to mAbs and cytokines. We arbitrarily defined “responding” lesions, those exhibiting a more than 1.5-fold change over two different controls (medium and IgG) in two independent biological readouts (out of 40 readouts measured, 35 were retained with a threshold of 95% of detected values). This stratification into responders (R) vs. non-responders (NR) enabled us to define in a retrospective manner which parameters expressed by peripheral or infiltrating T cells were essential for this response. Furthermore, we demonstrated the validity of the method by analyzing the predictive value of some parameters on retrospective clinical cohorts including 190 unresectable stage III−IV MMel patients.

### Study approval

Institutional review board approvals were granted by the University of Tübingen, the University of California, the University Hospital of Copenhagen, the University Hospital of Zürich, the University Hospital of Siena, the Sharett Institute of Oncology, the Aarhus University Hospital, the Centre Hospitalier de Nantes, and the Providence Cancer Center (for the ipilimumab-treated cohorts), the Laura & Isaac Perlmutter Cancer Center (for ipilimumab + nivolumab and nivolumab) and Gustave Roussy/Kremlin Bicêtre and Centre Hospitalier Lyon-Sud for the prospective cohort and retrospective cohorts (Supplementary Fig. 7d) which were previously described^68, 69^. The human study protocols were in accordance with the Declaration of Helsinki principles, and all patients provided informed consent before enrollment in the study.

### Prospective cohort of 37 patients

This cohort and its clinical parameters have been previously described^19^.

### Retrospective cohorts of 190 ipilimumab-treated patients

Patients enrolled in this study were from several centers: the University of Tübingen, University of Siena, University of California, University Hospital of Copenhagen, University Hospital of Zürich, Sharett Institute of Oncology, Aarhus University Hospital, Centre Hospitalier de Nantes, and the Providence Cancer Center. In all cohorts, blood samples were collected before injections of ipilimumab from patients participating in evaluations of ipilimumab as adjuvant therapy. Markers were assessed on PBMCs with the exception of the Sharett Institute of Oncology cohort (assessed on whole blood) after thawing. Patients’ characteristics can be found in Supplementary Table 2.

### Retrospective study on the adjuvant Phase II trial testing nivolumab + ipilimumab vs. nivolumab-treated patients

Information regarding this clinical trial can be found in reference^70^.

### PBMC and TILs preparations

Peripheral blood samples from patients or HV were carefully layered on top of a Ficoll-Hypaque density gradient media (PAA Laboratories). The ring of PBMC was collected and washed twice in PBS, resuspended in PBS, counted and stained for flow cytometric analyses or resuspended in CryoMaxx medium (PAA Laboratories) for storage in liquid nitrogen. Resected mLN specimens from MMel patients were placed in isotonic solution at least for 1 h. Next, tissue was cut and placed in dissociation medium, which consisted of RPMI1640, 1% penicillin/streptomycin (PEST, GIBCO Invitrogen), Collagenase IV (50 IU/ml), hyaluronidase (280 IU/ml), and DNAse I (30 IU/ml) (all from Sigma-Aldrich), and run on a gentle MACS Dissociator (Miltenyi Biotec) during 1 h. Cell samples were diluted in PBS, passed through a cell strainer and centrifuged for 5 min at 1500 r.p.m. Cells were finally resuspended in PBS, counted, stained for flow cytometric analyses or resuspended in CryoMaxx medium (PAA Laboratories) for storage in liquid nitrogen. All mLN included in the study were histologically confirmed to be invaded and patients enrolled in this prospective cohort were free of prior Abs-based immunotherapies.

### Ex vivo mLN assays

Dissociated cells from mLN were incubated in two 48-well plates at 0.3 × 10^6^/ml in complete medium (RPMI 1640 supplemented with 10% human AB serum [Institut de Biotechnologie Jacques Boy], 1% Penicillin/Streptomycin, 1% l-glutamine and 1% of sodium pyruvate [all from Gibco-Invitrogen]) and with isotype control, agonistic (CD137/CD137L) or antagonistic (PD-1/PD-L1, CTLA-4, Tim-3) mAbs or cytokines (IFNα2a [Roferon, ROF], IL-2) or their combinations (PD-1 + ROF, CTLA-4 + ROF, PD-1 + Tim-3, PD-1 + CTLA-4) as described in the Supplementary Fig. 1 and Supplementary Tables 1 and 7. After 18–24 h of incubation with or without drugs, cells were stimulated with PMA (5 ng/ml) (Sigma), ionomycin (125 ng/ml) (Sigma), and BD Golgi Stop (4 µl per 6 ml) (BD Biosciences) for 3−5 h. Cells were then collected, membrane stained to discriminate between different lymphocyte subsets (Supplementary Table 8), permeabilized with BD Cytofix/Cytoperm^TM^ kit (BD Biosciences). Intracellular staining was then performed using anti-IFNγ PE (BD Biosciences, clone B27) and anti-TNFα AF647 (BioLegend, clone Mab11) mAbs. For the second plate, after 4−5 days of culture with or without drugs, cells were collected and membrane stained, permeabilized with Foxp3/Transcription factor Fixation/Permeabilization kit (eBiosciences) and intranuclearly stained with anti-Ki67 PE (BD Biosciences, clone B56) and anti-Foxp3 APC (eBiosciences, clone PCH101) mAbs following the manufacturer’s recommendations. We arbitrarily defined “biological responses”, as those exhibiting a >1.5-fold increase over the values obtained with two negative controls (medium and Ig control mAb) in at least two independent biological readouts, except for CD4^+^FoxP3^+^ Treg for which a response was defined as a >1.5-fold decrease compared with the baseline levels in responders compared to non-responders.

### Flow cytometric analyses

For membrane labeling, PBMC and TILs were stained with fluorochrome-coupled mAbs (detailed in Supplementary Table 8), incubated for 20 min at 4°C and washed. Cell samples were acquired on a Cyan ADP 9-color (Beckman Coulter), BD FACS Canto II flow-cytometers or on an 18-color BD LSRII (BD Biosciences) with single-stained antibody-capturing beads used for compensation (Compbeads, BD Biosciences or UltraComp eBeads, eBiosciences). Data were analyzed with FlowJo software v7.6.5 or v10 (Tree Star, Ashland, OR, USA).

### Cytokine and chemokine measurements

Supernatants from cultured cells were monitored using the human Th1/Th2/Th9/Th17/Th22 13-plex RTU FlowCytomix Kit (eBiosciences), and human chemokine 6-plex kit FlowCytomix (eBiosciences) according to the manufacturer’s instructions and acquired on a Cyan ADP 9-color flow cytometer (Beckman Coulter). Analyses were performed by FlowCytomix Pro 3.0 Software (eBiosciences). Some measurements were performed by ELISA with IFNγ (BioLegend), IL-9 (BioLegend), TNFα (BD Biosciences), CCL2 (BD Biosciences), CCL3 (R&D Systems), CCL4 (R&D Systems), CCL5 (R&D Systems), and CXCL10 (BD Biosciences) kits in accordance with the manufacturer’s recommendations.

### Statistics

Data analyses were performed with the R software (http://www.R-project.org/). Graphical representations were performed either with R or Prism 5 (GraphPad, San Diego, CA, USA). For the “ex vivo metastatic lymph node (mLN) assay” 124 (blood) and 128 (tumor) parameters were analyzed and reported. The effectiveness of these biomarkers on resistance to CTLA-4 blockade was reported and graphed using the log transformed ratio between responders and non-responders or the area under the ROC curve (AUC statistic) on the *x*-axis and the Wilcoxon rank-sum test *p*-values on the *y*-axis. Distribution of biomarkers were also plotted through box and whiskers plots. Regarding the retrospective cohort evaluation, logistic regressions (univariate and multivariate) have been used to assess the association of covariates on binary endpoint (i.e., clinical response). Two survival endpoints were also evaluated: (i) overall survival (OS) was defined as the time from the date of sampling to death or the last follow-up, whichever occurred first and (ii) PFS was defined as the time from the date of sampling to death, disease progression or the last follow-up, whichever occurred first. For both survival endpoints (OS and PFS), survival curves were estimated using the Kaplan−Meier method by dichotomizing biomarkers through their median value or a chosen cutoff based on HV. The decision between median vs. a cutoff, is based on the fact that the PD-L1^+^CD8^+^ cells in most healthy volunteers were undetectable (with values close to 0), meaning that most of the patients would have been classified as supranormal. Therefore, we chose to calculate the median values for patients with respect to this parameter. In sharp contrast, the values of CD95^+^CD4^+^ cells in healthy volunteers were sizeable, allowing to estimate a threshold above which the patients could be considered supranormal. Cox models have been used to perform univariate and multivariate analysis. Graphical visualization of the effect of continuous biomarkers has been performed by modeling them through splines with 2 degrees of freedom. All the logistic and Cox models evaluated the biomarkers based on a continuous scale, were stratified on the centers, and adjusted for LDH, gender, age, tumor stage, CT, IT, and PKI as indicated in Supplementary Tables 3−6. In all cases, confidence intervals were reported at a nominal level of 95%.

### Data availability

An MTA was signed between Gustave Roussy and Laura and Isaac Perlmutter Cancer Center.

## Electronic supplementary material

Supplementary Information
