# Supplementary material for: Predictors of responses to immune checkpoint blockade in advanced melanoma
Source: Nat Commun. 2017 Sep 19;8:592. doi: 10.1038/s41467-017-00608-2 (PMC5605517; doi:10.1038/s41467-017-00608-2)
Supplement: Supplementary file 1 — Supplementary Information [file 41467_2017_608_MOESM1_ESM.pdf]

Description of Supplementary Files

File Name: Supplementary Information
Description: Supplementary Figures and Supplementary Tables

Supplementary Fig.1

Stimulation Condition			
1	Medium control		Medium control
2	mIgG1	9	αTIM-3
3	αCTLA-4	10	αTIM-3 + αPD1
4	αPD1	11	αPD-L1
5	αPD1 + αCTLA-4	12	IL-2
6	αCD137	13	IFN-α2a (Roferon)
7	αCD137L	14	IFNα2a + αCTLA-4
8	αCD137 + αCD137L	15	IFNα2a + αPD1

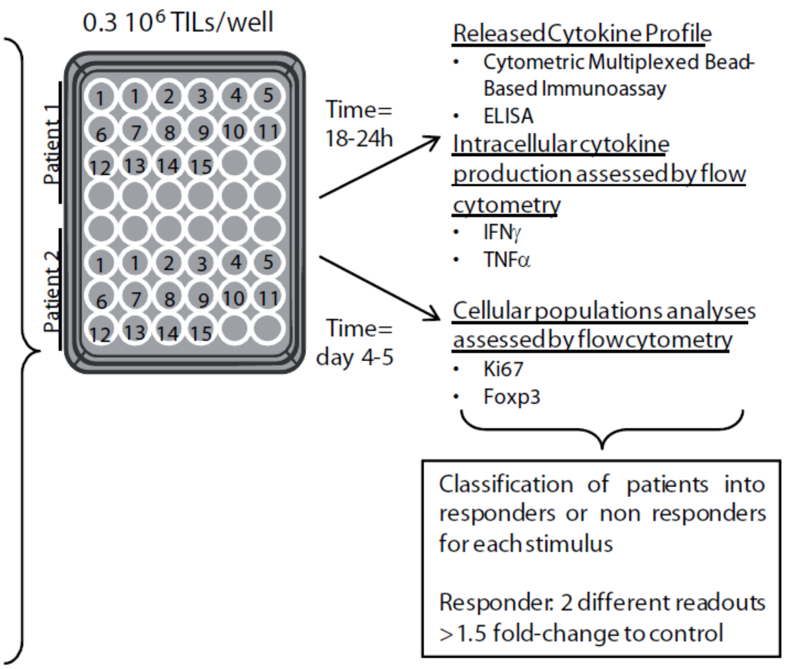

Supplementary Figure 1. Experimental setting of the « *ex vivo* mLN assay ». Metastatic lymph nodes (mLN) containing 4-98% melanoma tumor cells were resected, freshly mechanically and enzymatically dissociated using the Miltenyi Gentle MACs equipment for 1 hour at 37°C under rotation (2 incubation steps of 30 minutes). Whole cell suspensions were incubated in duplicate (one for the 18-24 h readout and one for the 4-5 day readout) wells at 0.3×10^6/ml with medium, versus isotype control mAb or a series of antagonistic or agonistic mAbs, combinations, or recombinant cytokines, as outlined. The *ex vivo* stimulation lasted 18-24 h (except in 2 cases where it lasted 48 h) before flow cytometric analyses of live $CD45^+$ cells, within $CD3^+CD4^+$, $CD3^+CD8^{+,}$ or $CD3^-CD56^+$ cell gates for intracellular staining of Th1 cytokines (IFNγ, TNFα) after a final 3-5hr activation with PMA ionomycin and GolgiStop. The 18-24 hr cytokine release was monitored by commercial ELISA or multiplex arrays. The day 4-5 timepoint was crucial for monitoring proliferation, by flow cytometric analyses of Ki67 on T, NK, and Treg populations.

Supplementary Fig. 2

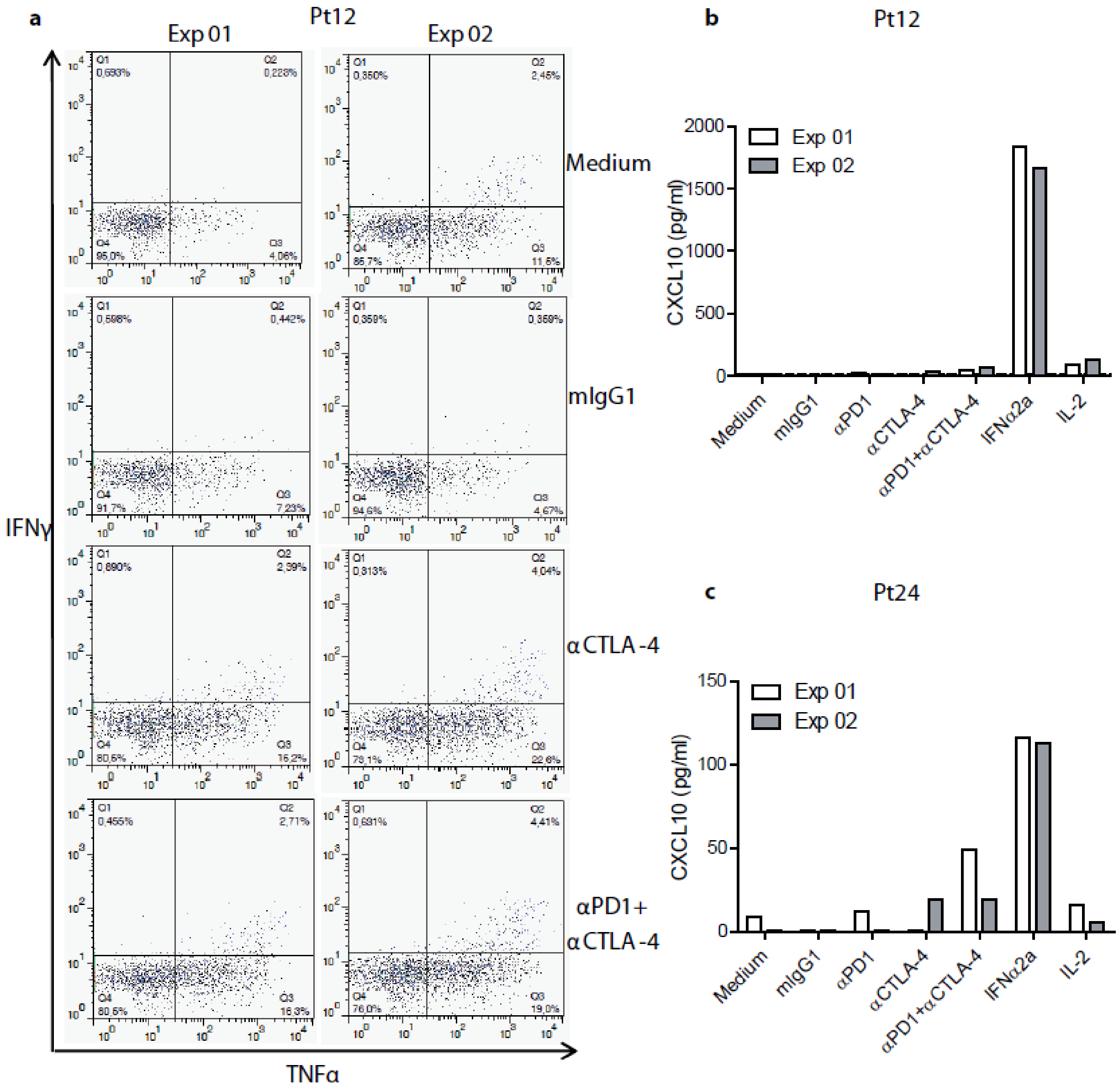

Supplementary Figure 2. Inter-individual variations in specimen handling and data harvesting in two patient lesions. Exemplification of positive scoring in several immunometrics for two patients using different *ex vivo* stimulations performed in parallel, independent experiments by the two first authors of the paper to cross-validate the findings and data mining approach. Flow cytometry dot plots are shown for the experiments performed by each individual on one patient (**a**), and CXCL10 dosage by ELISA performed by both individuals on two patient lesions (**b,c**).

Supplementary Fig.3

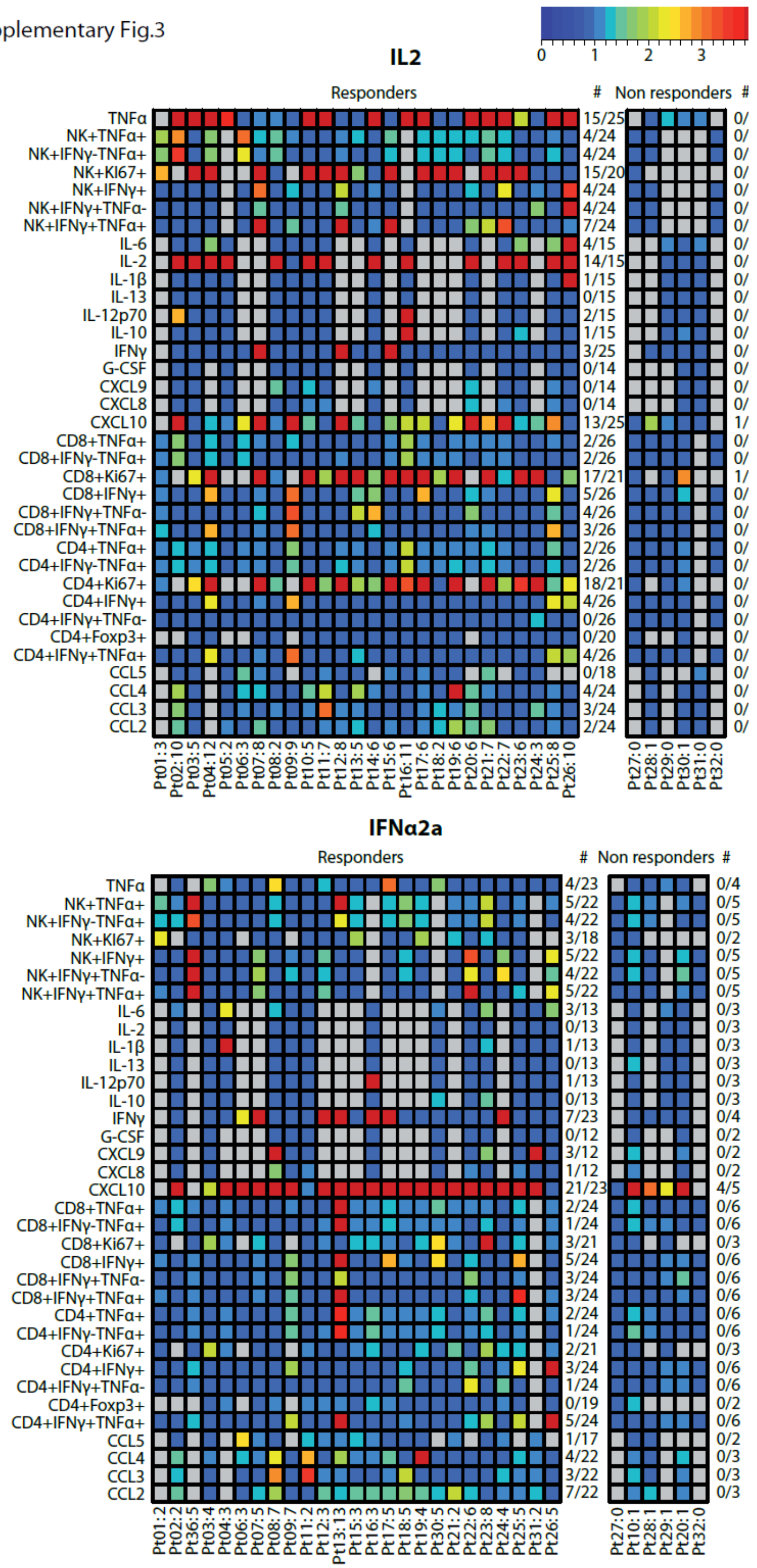

Supplementary Figure 3. Heatmaps segregating responding versus non-responding patient lesions in each stimulation axis (IL-2, IFNα2a). Heatmap depicting the immunometrics scoring in the IL-2 and IFNα2a stimulation axes. For improved readability of the scoring heatmap, patients are segregated according to their response lesions for the IL-2 and IFNα2a stimulation axes. Each column represents a patient and each row a parameter (an immunometric). Grey cells indicate that the marker could not be evaluated for the corresponding patient. The total sums of positive immunometrics (out of the number of evaluated immunometrics) are shown for each marker (y-axis) or patient (x-axis). The most representative data for each axis are plotted on a graph appearing in Fig. 1.

Supplementary Fig. 4

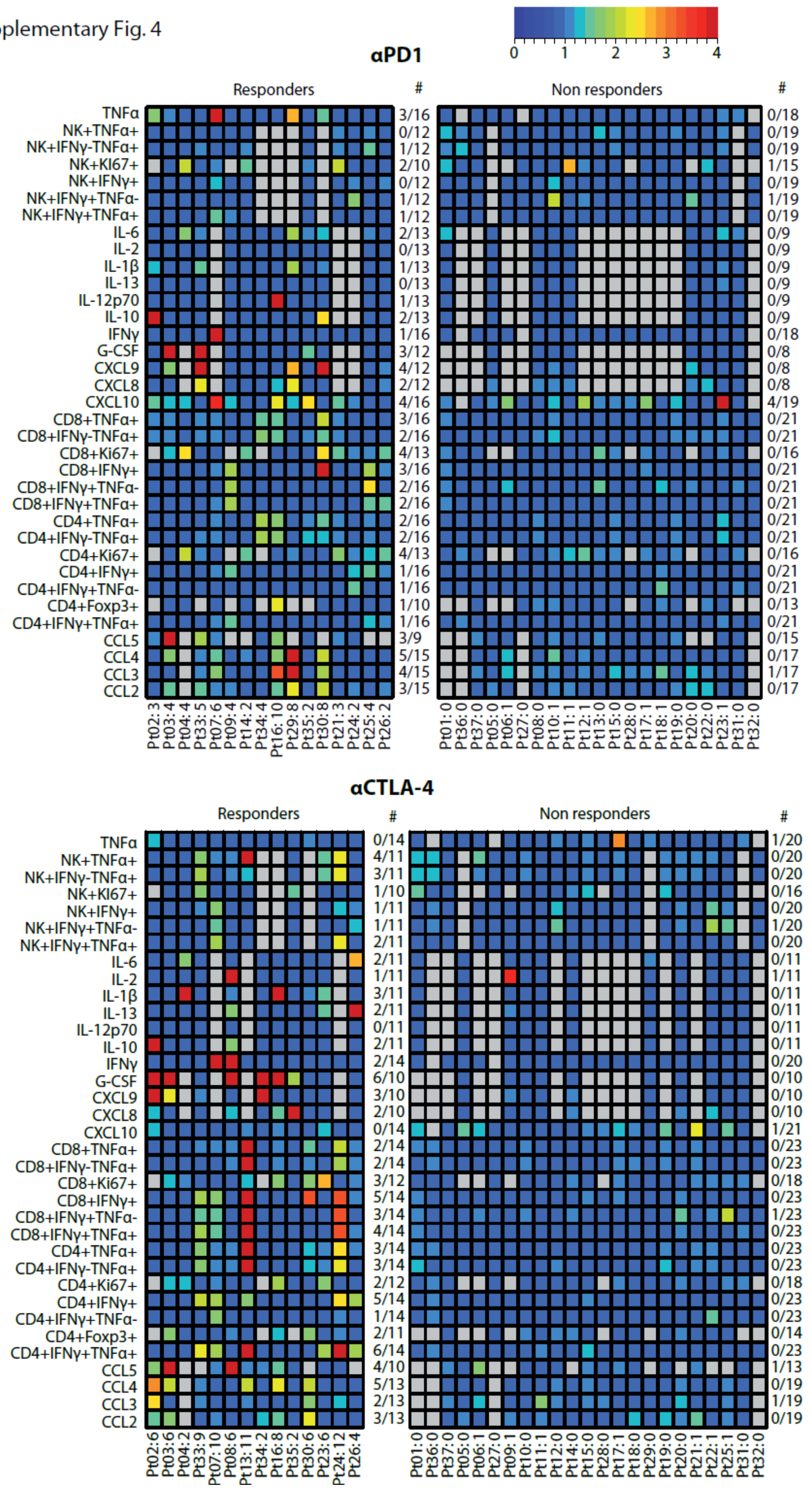

Supplementary Figure 4. Heatmaps segregating responding versus non-responding patient lesions in each stimulation axis (αPD-1, αCTLA-4). Heatmap depicting the immunometrics scoring in the αPD-1 and αCTLA-4 stimulation axes. Each column represents a patient and each row a parameter (an immunometric). Grey cells indicate that the marker could not be evaluated for the corresponding patient. The total sums of positive immunometrics (out of the number of evaluated immunometrics) are shown for each marker (y-axis) or patient (x-axis). The most representative data for each axis are plotted on a graph in Fig. 1.

Supplementary Fig. 5

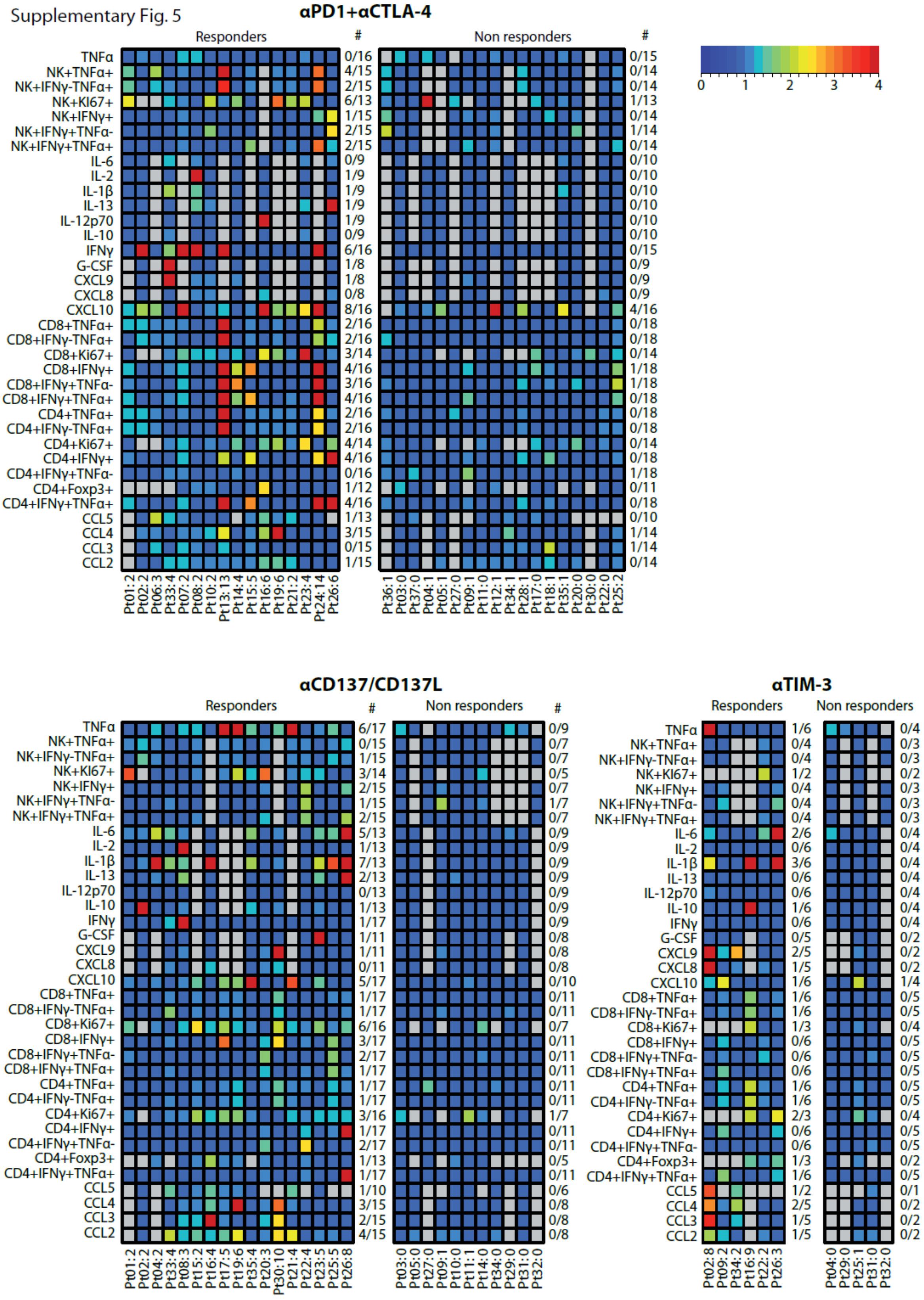

Supplementary Figure 5. Heatmaps segregating responding versus non-responding patient lesions in each stimulation axis (αCTLA-4+αPD-1, αCD137/CD137L, αTim-3). Heatmap depicting the immunometrics scoring in the αCTLA-4+αPD-1, αCD137/CD137L and αTim-3 stimulation axes. Each column represents a patient and each row a parameter (an immunometric). Grey cells indicate that the marker could not be evaluated for the corresponding patient. The total sums of positive immunometrics (out of the number of evaluated immunometrics) are shown for each marker (y-axis) or patient (x-axis). The most representative data for each axis are plotted on a graph appearing in Fig.1.

Supplementary Fig.6

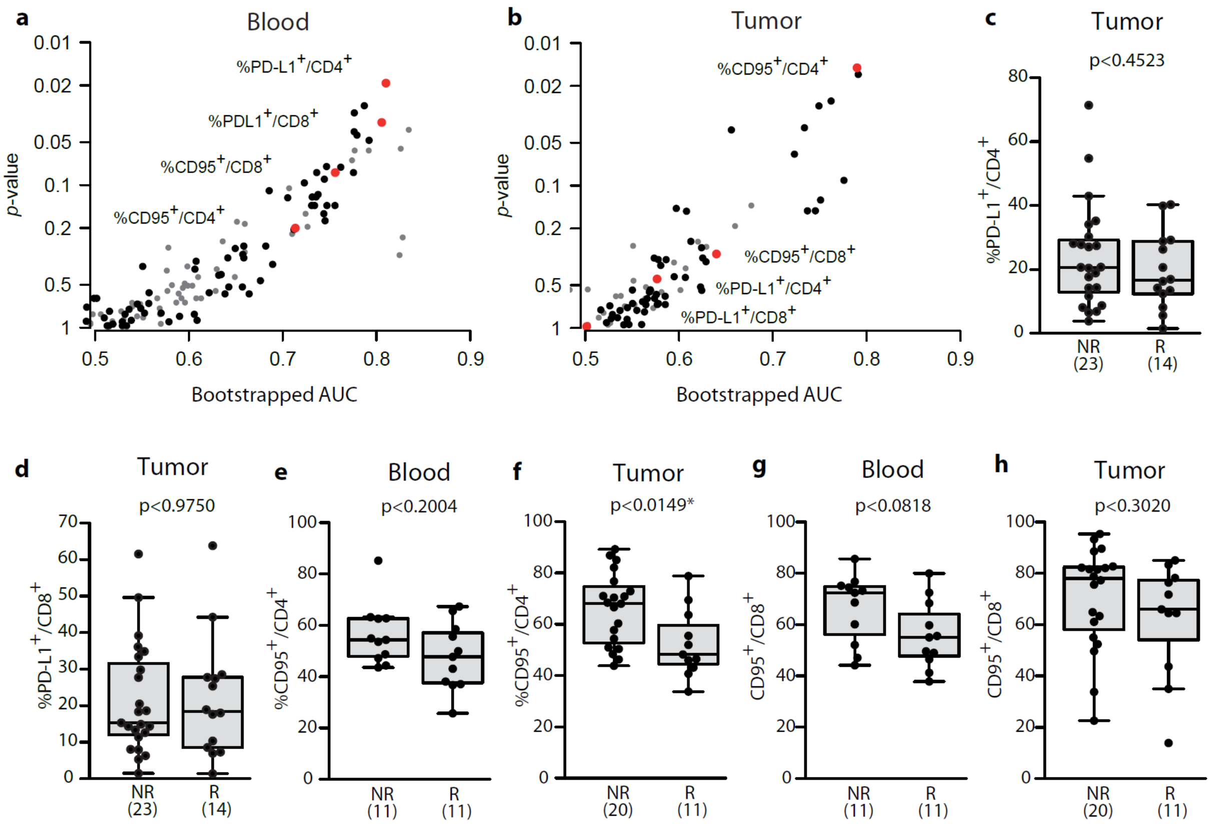

Supplementary Figure 6. PD-L1 expression on TILs does not predict resistance to CTLA-4 blockade with the "*ex-vivo* mLN assay". a-b. Display of the Wilcoxon rank sum test *p*-values versus the bootstrapped AUC of the markers used to assess the sensitivity to anti-CTLA-4+anti-PD-1 Abs in the blood (**a**) and tumor (**d**) in the *ex vivo* mLN assay. Each dot represents one marker; selected biomarkers are shown in red while biomarkers with low level of expression are shown in grey. **c-d**. Expression levels of PD-L1 on $CD4^+$ (**c**) and $CD8^+$ (**d**) TILs respectively in lesions responding (R) or not (NR) to the *ex vivo* mLN assay in the anti-CTLA-4 mAb stimulatory condition. **e-h.** Expression levels of CD95 on $CD4^+$ (**e-f**) and $CD8^+$ (**g-h**) in blood (**e, g**) or TILs (**f, h**) respectively in responding (R) or not (NR) lesions in the *ex vivo* mLN assay testing the anti-CTLA-4 mAb stimulatory condition. Each dot represents one patient. The absolute numbers of patients are indicated in both groups. *p*-values were obtained by Wilcoxon rank sum test. Box and whiskers plot are represented from the corresponding distribution (**c-h**).

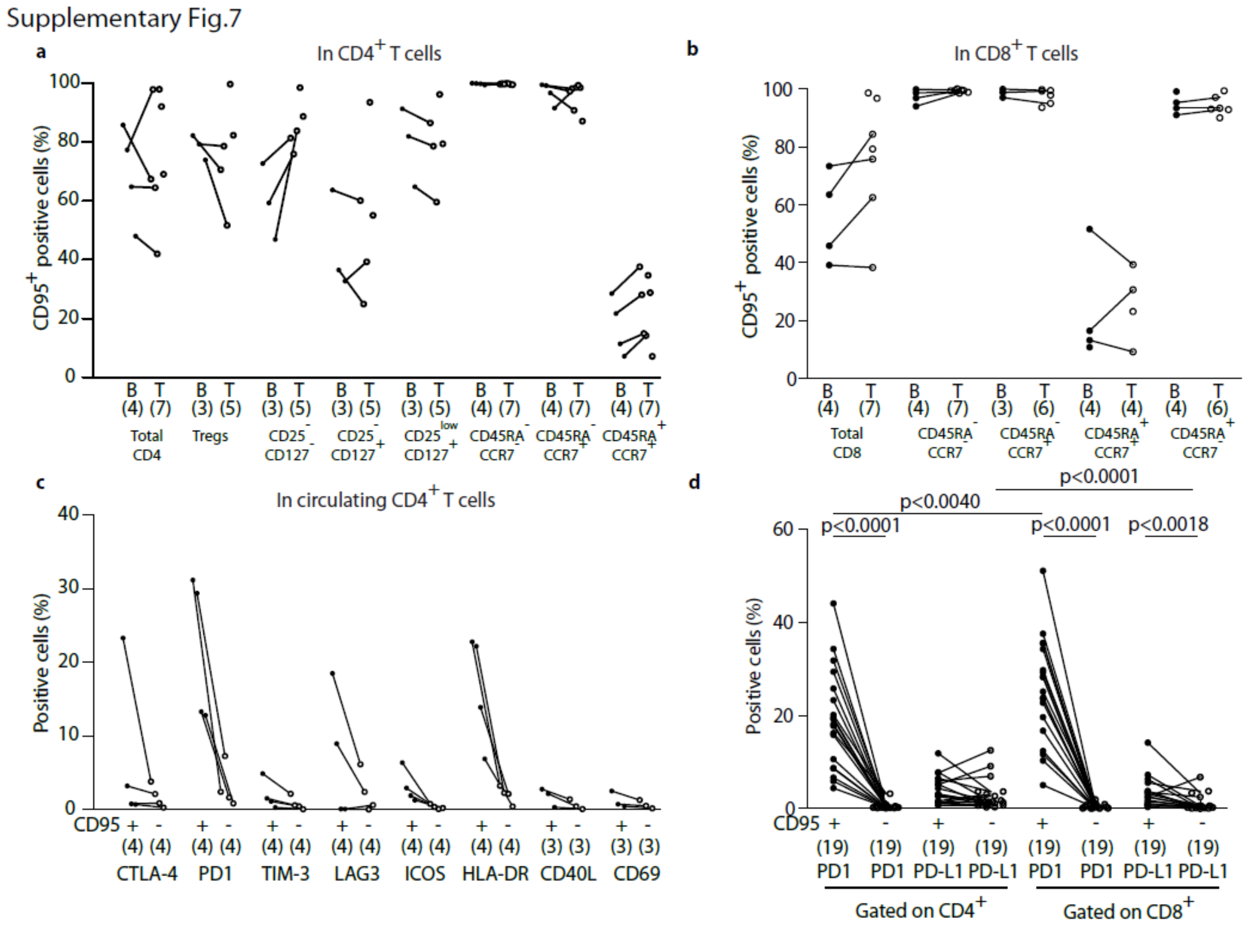

Supplementary Figure 7. CD95$^+$ T cells are effector memory cells expressing PD-1 and HLA-DR. a-b. Expression of CD95 on various CD4$^+$ (**a**) and CD8$^+$ (**b**) T cell subsets (defined using CD45RA, CCR7, CD127 and CD25 markers by flow cytometry analyses) in blood (B) and tumor (T) beds in 7 individuals diagnosed with stage III MMel. **c**. Expression of activation and exhaustion markers (indicated in the X axis) gating on CD95$^+$ (+) or CD95$^-$ (–) blood CD4$^+$ T cells in 7 individuals. **d**. CD95 expression according to PD1 and PD-L1 expression on circulating T cells. Each dot represents the value of one patient with the number of patients tested indicated in parentheses. *p*-values from Wilcoxon matched-pairs signed rank test are indicated.

Supplementary Fig.8

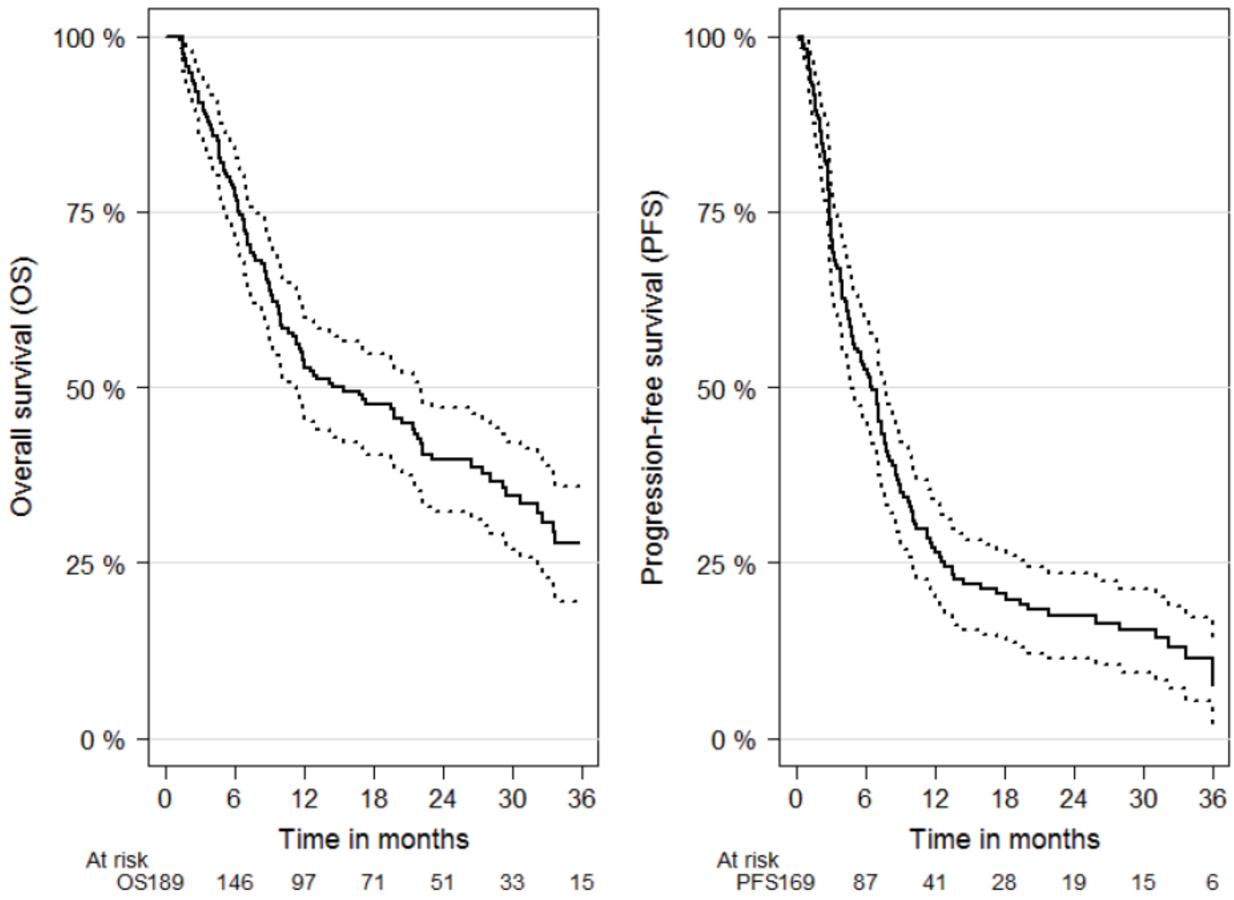

Supplementary Figure 8. PFS and OS in the 8 cohorts of MMel patients treated with ipilimumab and described in Supplementary Table 2.

Supplementary Fig.9

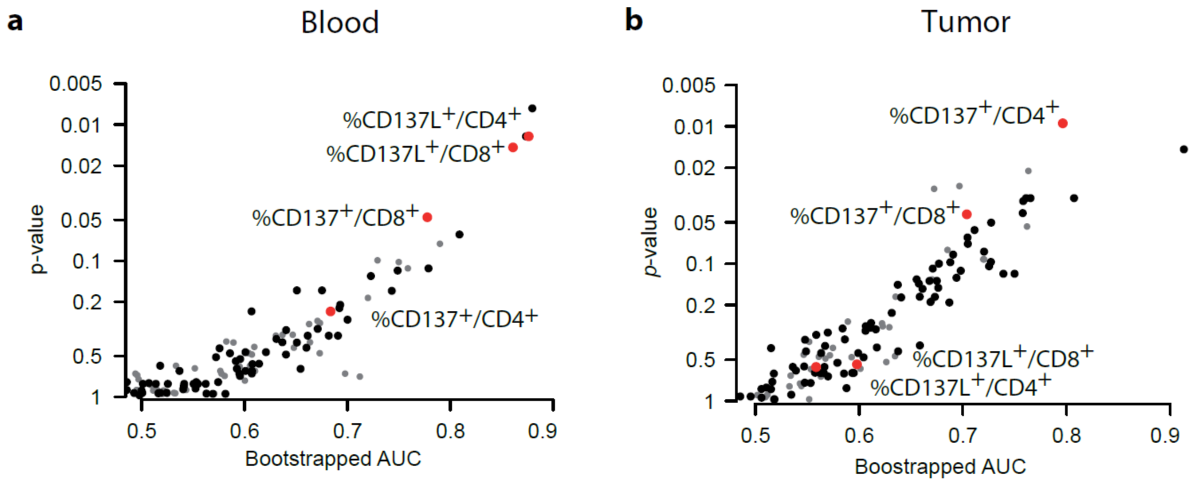

Supplementary Figure 9. CD137/CD137L expression on blood and tumor T cells are the best predictive markers for the *ex vivo* responses to the combination of anti-CTLA-4 + anti-PD-1 Ab. a and **b**. Display of the Wilcoxon rank sum test *p*-values versus the bootstrapped AUC of the markers used to assess the sensitivity to anti-CTLA-4+anti-PD-1 Abs in the blood (**a**) and tumor (**d**) in the *ex vivo* mLN assay. Each dot represents one marker; selected biomarkers are shown in red while biomarkers with low level of expression are shown in grey.

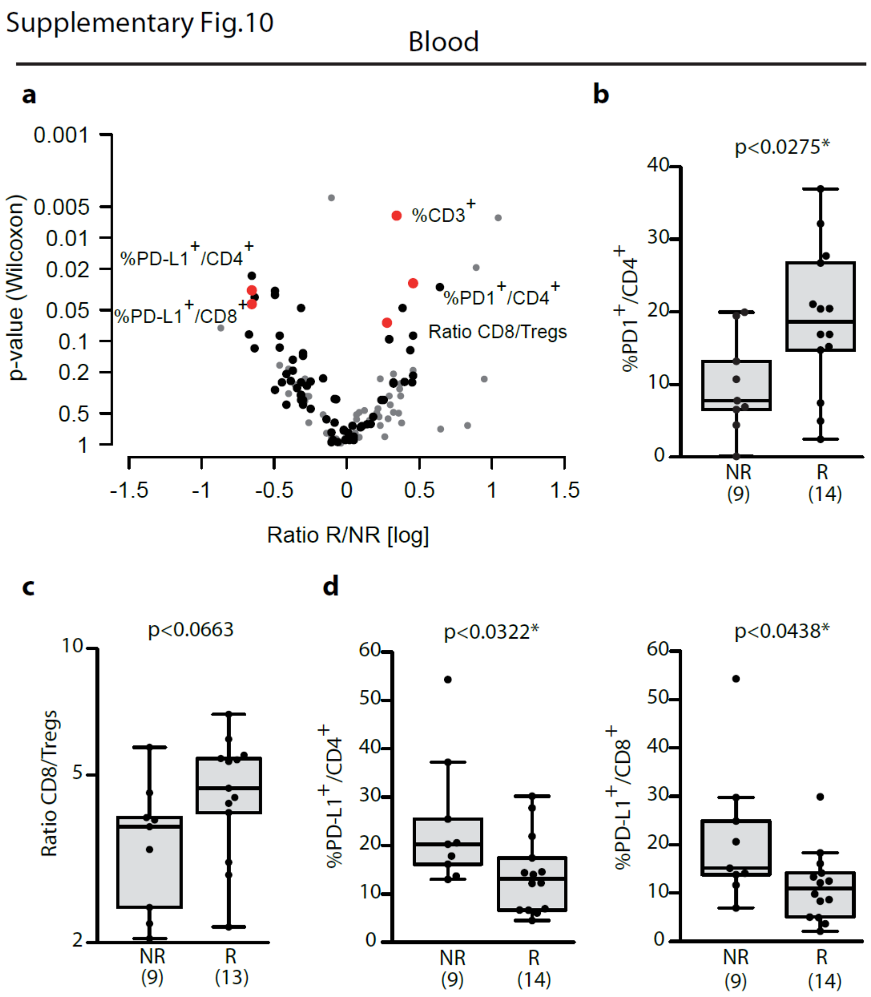

Supplementary Figure 10. PD-1 and PD-L1 expression on blood T cells and the CD8$^+$ T cell/Treg ratio in blood predict responses to αPD-1 mAb in *ex vivo* mLN assays. a. Display of the Wilcoxon rank sum test *p*-values versus the log transformed ratio between responders (R) and non responders (NR) to anti-PD-1 mAb in the blood. Each dot represents one marker; selected biomarkers are shown in red while biomarkers with low level of expression are shown in grey. **b-d**. Expression levels of PD-1 (**b**) on blood CD4$^+$ T cells, CD8/Treg ratio (**c**) and PD-L1 (**d**) on CD4$^+$ and CD8$^+$ T cells in patient lesions responding (R) or not (NR) to the *ex vivo* mLN assay in αPD-1 mAb stimulatory condition. Each dot represents one patient. The absolute numbers of patients are indicated in both groups. *p*-values obtained by Wilcoxon rank sum test are shown. Box and whiskers plot are represented from the corresponding distribution (**b-d**).

Supplementary Table 1. Detailed responses of patients treated with different ICB and cytokines.

	Stimulation in *ex vivo* mLN assays									
Patient	αCTLA-4	αPD1	αPD1+ αCTLA-4	αCD137 and/ or αCD137L	IFNα2a	IL-2	αPD1+αTIM-3	αTIM-3	αCTLA-4 + IFNα2a	αPD1 + IFNα2a
Pt01	-	-	+	+	+	+	n/d	n/d	+	+
Pt02	+	+	+	+	+	+	+	+	+	+
Pt03	+	+	-	-	+	+	+	n/d	-	-
Pt04	+	+	-	+	+	+	+	-	+	+
Pt05	-	-	-	-	n/d	+	-	n/d	n/d	n/d
Pt06	-	-	+	n/d	+	+	n/d	n/d	n/d	n/d
Pt07	+	+	+	n/d	+	+	n/d	n/d	n/d	n/d
Pt08	+	-	+	+	+	+	+	n/d	+	+
Pt09	-	+	-	-	+	+	n/d	+	n/d	+
Pt10	-	-	+	-	-	+	-	n/d	+	+
Pt11	-	-	-	-	+	+	-	n/d	+	+
Pt12	-	-	-	n/d	+	+	n/d	n/d	n/d	n/d
Pt13	+	-	+	n/d	+	+	n/d	n/d	n/d	n/d
Pt14	-	+	+	-	n/d	+	n/d	n/d	n/d	n/d
Pt15	-	-	+	+	+	+	n/d	n/d	n/d	n/d
Pt16	+	+	+	+	+	+	+	+	+	+
Pt17	-	-	-	+	+	+	n/d	n/d	n/d	n/d
Pt18	-	-	-	n/d	+	+	n/d	n/d	n/d	n/d
Pt19	-	-	+	+	+	+	n/d	n/d	n/d	n/d
Pt20	-	-	-	+	-	+	n/d	n/d	n/d	+
Pt21	-	+	+	+	+	+	n/d	n/d	n/d	n/d
Pt22	-	-	-	+	+	+	n/d	+	n/d	+
Pt23	+	-	+	+	+	+	+	n/d	+	+
Pt24	+	+	+	n/d	+	+	n/d	n/d	n/d	n/d
Pt25	-	+	-	+	+	+	n/d	-	n/d	+
Pt26	+	+	+	+	+	+	n/d	+	n/d	+
Pt27	-	-	-	-	-	-	-	n/d	-	-
Pt28	-	-	-	n/d	-	-	n/d	n/d	n/d	n/d
Pt29	-	+	n/d	-	-	-	-	-	-	-
Pt30	+	+	-	+	+	-	n/d	n/d	+	+
Pt31	-	-	n/d	-	+	-	n/d	-	+	-
Pt32	-	-	n/d	-	-	-	n/d	-	-	-
Pt33	+	+	+	+	n/d	n/d	n/d	n/d	n/d	n/d
Pt34	+	+	-	-	n/d	n/d	n/d	+	n/d	n/d
Pt35	+	+	-	+	n/d	n/d	n/d	n/d	n/d	n/d
Pt36	-	-	-	n/d	+	n/d	n/d	n/d	+	+
Pt37	-	-	-	n/d	n/d	n/d	n/d	n/d	n/d	n/d
Total R/NR (% of R)	14/23 (37.8)	16/21 (43.2)	16/18 (47.1)	17/11 (60.7)	25/6 (80.6)	26/6 (81.2)	6/5 (54.5)	6/5 (54.5)	11/4 (73.3)	15/5 (75)

n/d : not done

Supplementary Table 2: Cohorts and patient's characteristics

	Overall (190)	CA (19)	CH (16)	DE (3)	DK (67)	FR (15)	IT (10)	OR (20)	JE (40)
Gender									
Female	93 (49%)	8 (42%)	5 (31%)	0 (0%)	36 (54%)	12 (80%)	4 (40%)	9 (45%)	19 (47%)
Male	97 (51%)	11 (58%)	11 (69%)	3 (100%)	31 (46%)	3 (20%)	6 (60%)	11 (55%)	21 (53%)
Age									
Mean (SD)	61 (13)	64 (12)	58 (15)	64 (11)	63 (12)	66 (14)	56 (16)	59 (13)	58 (14)
LDH									
Low	112 (62%)	6 (32%)	13 (100%)	1 (33%)	56 (85%)	11 (73%)	2 (22%)	6 (30%)	17 (47%)
High	69 (38%)	13 (68%)	0 (0%)	2 (67%)	10 (15%)	4 (27%)	7 (78%)	14 (70%)	19 (53%)
Missing	9	0	3	0	1	0	1	0	4
Tumor stage									
III	13 (7%)	1 (5%)	2 (12%)	0 (0%)	0 (0%)	8 (53%)	1 (10%)	1 (5%)	0 (0%)
IV	177 (93%)	18 (95%)	14 (88%)	3 (100%)	67 (100%)	7 (47%)	9 (90%)	19 (95%)	40 (100%)
Tumor response									
PD	127 (67%)	11 (58%)	13 (81%)	3 (100%)	42 (63%)	2 (13%)	6 (60%)	17 (85%)	33 (83%)
SD	31 (16%)	1 (5%)	1 (6%)	0 (0%)	14 (21%)	7 (47%)	4 (40%)	0 (0%)	4 (10%)
PR	18 (9%)	6 (32%)	0 (0%)	0 (0%)	9 (13%)	2 (13%)	0 (0%)	1 (5%)	0 (0%)
CR	14 (7%)	1 (5%)	2 (12%)	0 (0%)	2 (3%)	4 (27%)	0 (0%)	2 (10%)	3 (8%)
Previous CT									
Yes	71 (42%)	9(47%)	8 (50%)	2 (67%)	11 (16%)	3 (20%)	8 (80%)	0 (0%)	30 (75%)
Missing	20	0	0	0	0	0	0	20	0
Previous IT									
Yes	66 (35%)	4 (21%)	1 (6%)	1 (33%)	19 (28%)	11 (73%)	3 (30%)	14 (70%)	13 (32%)
Previous PKI									
Yes	16 (9%)	2 (11%)	3 (19%)	1 (33%)	3 (4%)	1 (7%)	3 (30%)	0 (0%)	3 (8%)
Missing	20	0	0	0	0	0	0	20	0
Ipilimumab dose									
3 mg/kg	143 (88%)	0 (0%)	16 (100%)	3 (100%)	67 (100%)	15 (100%)	10 (100%)	20 (100%)	12 (100%)
10 mg/kg	19 (12%)	19 (100%)	0 (0%)	0 (0%)	0 (0%)	0 (0%)	0 (0%)	0 (0%)	0 (0%)
Missing	28	0	0	0	0	0	0	0	28
Co-treatment									
GMCSF	19 (10%)	19 (100%)	0 (0%)	0 (0%)	0 (0%)	0 (0%)	0 (0%)	0 (0%)	0 (0%)
IL2	3 (2%)	0 (0%)	0 (0%)	3 (100%)	0 (0%)	0 (0%)	0 (0%)	0 (0%)	0 (0%)

PD: progression disease, SD: stable disease, PR: partial response, CR: complete response, CT: chemotherapy and/or radiation, IT: immunotherapy, PKI: protein kinase inhibitors

Supplementary Table 3: Clinical prognostic parameters for Ipilimumab responses (PD vs SD+PR+CR), OS and PFS

	Tumor response	Overall survival	Progression - free survival
Age	1.002<br>[0.997 ; 1.007]<br>P = 0.468	0.992<br>[0.977 ; 1.006]<br>P = 0.268	0.992<br>[0.979 ; 1.006]<br>P = 0.253
Gender<br>(ref = 'female')	0.94<br>[0.83 ; 1.07]<br>P = 0.38	1.09<br>[0.76 ; 1.58]<br>P = 0.63	1.26<br>[0.89 ; 1.77]<br>P = 0.20
LDH<br>(ref = 'low')	0.87<br>[0.74 ; 1.01]<br>P = 0.068	2.31<br>[1.47 ; 3.62]<br>P < 0.001	1.51<br>[0.96 ; 2.38]<br>P = 0.073
Previous CT<br>(ref = 'no')	0.92<br>[0.79 ; 1.08]<br>P = 0.32	1.62<br>[1.02 ; 2.58]<br>P = 0.043	1.01<br>[0.68 ; 1.48]<br>P = 0.97
Previous IT<br>(ref = 'no')	1.08<br>[0.94 ; 1.25]<br>P = 0.27	0.71<br>[0.47 ; 1.08]<br>P = 0.11	0.77<br>[0.53 ; 1.13]<br>P = 0.18
Previous PKI<br>(ref = 'no')	0.74<br>[0.58 ; 0.93]<br>P = 0.012	2.04<br>[1.01 ; 4.09]<br>P = 0.046	1.95<br>[1.04 ; 3.67]<br>P = 0.038
Tumor stage<br>(ref = 'stage III')	1.03<br>[0.77 ; 1.40]<br>P = 0.83	1.01<br>[0.29 ; 3.47]<br>P = 0.99	0.55<br>[0.21 ; 1.43]<br>P = 0.22

All models were stratified on the center. Odds Ratio (for the tumor response) and Hazard Ratio (for the survival endpoints) with 95% confidence intervals. CT: chemotherapy and/or radiation, IT: immunotherapy, PKI: protein kinase inhibitors.

Supplementary Table 4: Association between CD95 and PD-L1 (continuous scale) and the ipilimumab responses (PD vs SD+PR+CR)

Model	CD95.CD4	CD95.CD8	PDL1.CD4	PDL1.CD8
Univariate	0.978<br>[0.959 ; 0.997]<br>P = 0.023	0.992<br>[0.973 ; 1.011]<br>P = 0.40	0.996<br>[0.959 ; 1.034]<br>P = 0.84	0.977<br>[0.947 ; 1.006]<br>P = 0.13
Stratify on center	0.979<br>[0.958 ; 1.001]<br>P = 0.060	1.007<br>[0.984 ; 1.032]<br>P = 0.56	0.995<br>[0.941 ; 1.053]<br>P = 0.87	0.972<br>[0.924 ; 1.020]<br>P = 0.25
Stratify on center Adjust for LDH, gender, age, tumor stage, CT, IT and PKI	0.980<br>[0.955 ; 1.005]<br>P = 0.12	1.000<br>[0.968 ; 1.031]<br>P = 0.98	0.963<br>[0.893 ; 1.033]<br>P = 0.31	0.937<br>[0.869 ; 1.001]<br>P = 0.068

Odds ratios and 95% confidence intervals. Final model in grey.

Supplementary Table 5: Association between CD95 and PD-L1 (continuous scale) and the progression free- and overall survivals

Progression free survival				
Model	CD95.CD4	CD95.CD8	PDL1.CD4	PDL1.CD8
Univariate	1.007<br>[0.997 ; 1.017]<br>P = 0.19	0.996<br>[0.986 ; 1.006]<br>P = 0.44	1.021<br>[0.999 ; 1.043]<br>P = 0.057	1.021<br>[1.001 ; 1.042]<br>P = 0.040
Stratify on center	1.001<br>[0.989 ; 1.012]<br>P = 0.92	0.998<br>[0.986 ; 1.009]<br>P = 0.69	1.022<br>[0.993 ; 1.051]<br>P = 0.15	1.016<br>[0.986 ; 1.046]<br>P = 0.30
Stratify on center<br>Adjust for LDH, gender, age, tumor stage, CT, IT and PKI	1.004<br>[0.991 ; 1.017]<br>P = 0.59	0.999<br>[0.984 ; 1.015]<br>P = 0.93	1.044<br>[1.011 ; 1.079]<br>P = 0.009	1.032<br>[0.999 ; 1.065]<br>P = 0.056
**Overall Survival**				
Model	CD95.CD4	CD95.CD8	PDL1.CD4	PDL1.CD8
Univariate	1.010<br>[0.999 ; 1.022]<br>P = 0.082	1.013<br>[1.001 ; 1.025]<br>P = 0.031	1.036<br>[1.012 ; 1.059]<br>P = 0.003	1.043<br>[1.023 ; 1.063]<br>P < 0.001
Stratify on center	1.009<br>[0.996 ; 1.022]<br>P = 0.16	1.001<br>[0.989 ; 1.014]<br>P = 0.83	1.027<br>[0.993 ; 1.062]<br>P = 0.12	1.045<br>[1.015 ; 1.076]<br>P = 0.003
Stratify on center<br>Adjust for LDH, gender, age, tumor stage, CT, IT and PKI	1.010<br>[0.994 ; 1.027]<br>P = 0.21	1.009<br>[0.991 ; 1.028]<br>P = 0.33	1.041<br>[0.995 ; 1.089]<br>P = 0.081	1.053<br>[1.012 ; 1.096]<br>P = 0.011

Hazard ratios and 95% confidence intervals. Final model in grey.

Supplementary Table 6: Association between CD95/CD4 (>70 vs. ≤70) and overall survival

	Hazard Ratio	95% CI	p-value
Expression of CD95/CD4 (> 70 vs. ≤70)	1.96	[1.10 - 3.48]	0.022
LDH status	2.61	[1.51 - 4.52]	0.001
Age	0.979	[0.963 - 0.996]	0.018
Gender	0.90	[0.58 - 1.42]	0.66
Tumor stage	0.61	[0.11 - 3.29]	0.56
Previous CT	1.36	[0.78 - 2.39]	0.28
Previous IT	0.76	[0.46 - 1.24]	0.27
Previous PKI	1.33	[0.60 - 2.95]	0.48

CI: confidence interval. Adjusting covariates are in italic

Supplementary Table 7: Reagents used for mLN *ex-vivo* assay

Stimulation Condition	Clone	Source	Final Conc.
Medium control			
mIgG1	11711	R&D	10µg/ml
anti-PD-1	PD1m.3	Dr. Chen's lab	10µg/ml
anti-PD-L1	5H1	Dr. Chen's lab	
anti-Tim-3	2E2	Dr. Kuchroo's lab	10µg/ml
anti-CTLA-4	BMS-734016	Yervoy	10µg/ml
anti-CD137	6B4	Dr. Choi's lab	5µg/ml
anti-CD137L	5F4	Dr. Choi's lab	5µg/ml
IL-2		(Proleukine ®) Norvartis	100 IU/ml
IFN-α2A		(Roferon®) Roche Pharma	1000 IU/ml

Supplementary Table 8: List of monoclonal antibodies for flow cytometry

Name	Fluorochrome	Company	Reference	Clone
CD8	FITC	BD	555366	RPA -T8
CD4	PerCP	BD	345770	SK3
CD56	PE Cy7	Beckman	A21692	N901
CD3	VioBlue	Miltenyi	130-094-363	OKT3
Dead cells	Yellow	Invitrogen	L34957	-
CD45	APC AF750	Beckman	A79392	J.33
Tim -3	APC	eBiosciences	17-3109-42	F38-2E2
CD152 (CTLA - 4)	PE	BD	555853	BNI3
CD137 (CD137)	APC	Biolegend	309810	4B4-1
CD137L (CD137L)	PE	BD	559446	C65-485
CD274 (PD -L1)	APC	Biolegend	329708	29E.2A3
CD95 (Fas)	APC	BD	558814	DX2
CD95 (Fas)	PE	BD	555674	DX2
CD178 (FasL)	PE	eBiosciences	12-9919-42	NOK -1
CD69	APC	BD	555531	FN50
CD69	PerCP	BioLegend	310928	FN50
CD25	PE	BD	555432	M-A251
CD45RA	PE	BD	555489	HI100
CD314 (NKG2D)	PE	Miltenyi	130-092-672	BAT221
CD27	PE	BD	555441	M-T271
CD279 (PD - 1)	PE Cy7	Beckman	A78885	PD-1.3.5
CD27	APC	BD	558664	M-T271
CD127	APC	Miltenyi	130-094-890	MB15 -18C9
LAG3	FITC	R&D	FAB2319F	Polyclonal Ab
CD14	FITC	BD	555397	M5E2
CD15	PB	BioLegend	323022	W6D3
HLA DP/DR/DQ	APC	Miltenyi	130-104-824	REA332
HLA - DR	Pacific Blue	Beckman	A74781	Immu-357
CD11c	PE Cy7	BioLegend	301608	3.9
CD11b	PE Cy7	BD	557743	ICRF44
CD19	PE Cy7	BD	557835	SJ25C1
CD20	PE	Miltenyi	130-091-109	LT20
TNFα	AF647	Biolegend	502916	Mab11
IFNγ	PE	BD	559327	B27
Ki67	PE	BD	556027	B56
Foxp3	APC	eBiosciences	17-4776-42	PCH101
ICOS	PE	BD	557802	DX29
CCR7	BV421	BioLegend	353208	G043H7
CD40L	FITC	BD	555699	TRAP1
